# Economic evaluations in the palliative and end-of-life care settings: A systematic review of existing evidence, methods and quality

**DOI:** 10.1177/02692163261418546

**Published:** 2026-02-25

**Authors:** Claudia Fischer, Elisabeth Saly, Michael Berger, Eva Katharina Masel, Judit Simon

**Affiliations:** 1 Department of Health Economics, Center for Public Health, Medical University of Vienna, Austria; 2 Department of Internal Medicine I, Division of Palliative Care, Medical University of Vienna, Austria

**Keywords:** cost-effectiveness analysis, economic evaluation, end-of-life care, palliative care, resource allocation, systematic review, terminal care

## Abstract

**Background:**

Rising need for palliative and end-of-life care requires reliable cost-effectiveness evidence to support optimal resource allocation. Relevant value propositions and the applicability of conventional economic evaluation methods, however, may differ from other healthcare fields.

**Aim:**

To synthesise and critically appraise context-specific economic evaluations with comprehensive methodological and quality lenses including decision-making aspects.

**Design:**

We conducted a systematic review of published palliative and end-of-life economic evaluations following a registered, peer-reviewed protocol (CRD42020148160). Cost-effectiveness results, methods, reporting quality (CHEERS), study quality (CHEC) and decision-making contexts were summarised narratively.

**Data sources:**

The databases EMBASE, HTA-Database, MEDLINE, and NHS-EE-Database were searched between 2010 and 2024.

**Results:**

Of the 4190 identified references, 46 studies were included. Overall, 59% of the studies stemmed from four countries (UK, Canada, the Netherlands, USA), 54% were trial-based economic evaluations, 59% investigated cancer-related interventions, 41% were conducted in hospital settings, 63% were cost-utility analyses with 83% using EQ-5D for QALY-calculations. Studies typically took a health (and social) care perspective (63%) with 58% corresponding to national health technology assessment decision-making requirements. Of the evaluated interventions, 51% were cost-effective. Reporting quality (52%–96%) and study quality (56%–94%) greatly varied.

**Conclusions:**

Economic evaluations in palliative and end-of-life care settings mainly adhered to commonly required decision-making frameworks. This may result in sub-optimal analytical perspectives leading to important missed consequences, omitted alternative value considerations, and ignorance of some existing context-specific methodological recommendations. Developing and promoting consensus-based, context-specific methodological recommendations would be crucial to enhance the appropriateness of economic evaluation evidence in this context.

**What is already known about the topic?**

- Value propositions and the applicability of conventional economic evaluation methods for palliative and end-of-life care may differ from other healthcare fields
- Methodological challenges and relevant optimisation suggestions were summarised in a previous systematic review
- To date, it is unclear how applied methods of economic evaluations of palliative and end-of-life care interventions address these challenges

**What this paper adds**

- This systematic review assesses comprehensively the results, methods, reporting and study qualities and decision-making aspects of the existing economic evaluation literature in the palliative and end-of-life care fields.
- Most studies were conducted from a health and social care perspective as per national decision-making requirements, varied in reporting and study qualities, were heterogenous in terms of diseases, populations and interventions, and mainly focussed on hospital care from high-income countries.
- Applied economic evaluation methods remain sub-optimal concerning outcomes, perspectives and applied value frameworks.

**Implications for practice, theory or policy**

- Current cost-effectiveness evidence is not reflective of the setting diversity of palliative and end-of-life interventions; and studies from low- and middle-income countries are extremely limited
- The suitability of conventional decision-making methods and the QALY framework for palliative and end-of-life care should be revisited alongside emerging evidence for different context-specific societal preferences.
- Organisations and policy makers responsible for decision-making should consider expanding and optimising their guidelines for palliative and end-of-life economic evaluations.

## Introduction

The continuing growth of the number of people living with incurable and life limiting conditions corresponds with a surge in demand for palliative and end-of-life care.^1^ Global estimations predict 56.8 million individuals in need of palliative care every year, 30% of them due to cancer.^2^ This escalating demand places a significant strain on healthcare budgets.^3–5^ Health and care services for advanced cancer are particularly costly,^5^ with inpatient care and drug expenditure being responsible for the highest proportion.^6^

While curative care aims to overcome disease and promote recovery with overall quality-of-life improvement, palliative and end-of-life care aims to relieve the suffering of patients and their families using a holistic approach.^7^ Palliative and end-of-life care interventions hold promise for improving the quality, appropriateness and benefit of care. The Lancet Oncology Commission on integration of oncology and palliative care^8^ and the American Society of Clinical Oncology^9^ recommend that palliative care services are integrated early in the disease course, alongside active treatment.

Patients receiving palliative care represent a highly heterogeneous group, differing in age, illness type and disease stage, each with distinct care needs.^10^ Health and care services are provided in various settings, so treatment paths can differ greatly.^11,12^ A significant proportion of annual health expenditure is allocated to the less than 1% of people who die each year, especially in high-income countries.^13^ Furthermore, growing evidence points to potentially excessive treatments, which are not considered beneficial for patients near the end of life.^13–15^ Conversely, in low-and-middle-income countries, disparities in access to appropriate end-of-life care often arise due to financial inequities.^13^ As a result, there is a growing need to systematically assess the benefits and costs of palliative and end-of-life care interventions and services.^16,17^ High-quality economic evidence can support fair and transparent decisions about how to allocate limited healthcare resources to maximise benefit for patients.^18^ However, such evidence is notably scarce in the context of palliative and end-of-life care.^19,20^ Beyond known limitations in funding and policy interest, as well as ethical concerns regarding the explicit valuation of human life, this scarcity can also be attributed to the numerous, complex methodological challenges in the context of palliative and end-of-life care research.^21–23^ These include practical challenges such as the difficulty of including vulnerable patients in research and high attrition rates inherent to the end-of-life care setting.

Methodological challenges identified in an earlier review include ambiguity in the selection and measurement of outcomes, non-standardised measurement and valuation of costs, and difficulties in reliable, preference-based outcome valuation.^21^ Palliative care usually involves complex, multidimensional interventions that focus on improving the quality of life of people with serious, life-limiting illnesses and their families.^24^ These interventions often include not only medical treatments but also psychological, social, emotional and spiritual support, and vary greatly depending on the needs of the patients, their carers and the setting in which care is provided.^24,25^ As a result, economic evaluations in this area must take into account the complex nature of care, considering both direct and indirect cost impacts, as well as the broad benefits to patients and families.^24^ The complexity makes it difficult to quantify the impact and value of specific components of care in economic evaluations.^25^ Furthermore, there is an ongoing debate as to whether conventional economic evaluation methods based on the quality-adjusted life year (QALY) framework are sufficiently capable of capturing the profound impact of end-of-life care interventions.^26–30^ The Palliative Care Yardstick (PalY) is a proposed modified QALY framework that incorporates additional dimensions, such as spillover effects or components of a ‘good death’.^30^ Conventional health economic outcome measures such as the EQ-5D were found to be inadequate in addressing all aspects of quality of life relevant to individuals in this population, such as spirituality or preparation for death.^31–33^ An alternative outcome measure for the end-of-life context would be the more recently developed ICECAP Supportive Care Measure (ICECAP-SCM),^34^ that focuses on the broader wellbeing based on the capability concept by Sen and Nussbaum.^35,36^ However, it remains to be seen when and how such capability measures are used in practice.^37^

To accommodate potentially higher societal value preferences for the end-of-life context or conditions with major quality-of-life limitations, some health technology assessment organisations have included value modifiers that give additional weight to improvements in the health of individuals with more severe health conditions.^38,39^ However, it remains unclear whether such modifiers accurately reflect societal preferences. Empirical studies examining the value society places on life extension for palliative and end-of-life care patients have yielded inconsistent results.^40–42^

The absence of comprehensive, context-specific considerations in existing guidance for economic evaluation methods exacerbates the underlying research challenges. Earlier recommendations include the necessity to develop robust methods for assessing outcomes, accounting for evolving patient preferences over time, weighing the implications of decisions on equitable care distribution, and adopting a societal costing perspective.^43^ Nevertheless, despite these recommendations, the methodologies for economic evaluations in palliative and end-of-life care have not significantly evolved during the 2010s.^19^ Consequently, economic evaluations assessed earlier varied substantially, presenting challenges to their comparability and validity.^23,44^

Since then, relatively recent developments call for an updated review of economic evidence in the palliative and end-of-life care settings. First, new outcome measures have been developed, translated and/or further validated.^34,45,46^ Second, multiple organisations responsible for decision-making have introduced severity modifiers.^47^ Third, the potential inclusion of caregiver and family spillover effects has been more frequently included in decision-making guidelines.^48–50^ Finally, the overall importance and visibility of palliative and end-of-life care have increased, also due to its relevance to new emerging national policies about assisted suicide globally.^19,51,52^

The objective of this systematic literature review was to comprehensively identify and thoroughly analyse latest developments in the conduct of economic evaluations of palliative and end-of-life care interventions. Besides synthesising overall cost-effectiveness evidence, we also assessed applied methods in detail alongside reporting and study qualities in light of the given decision-making context. This aims to enhance the robustness and reliability of future evidence in this field.

## Methods

### Review design

This protocol-based systematic review of economic evaluations is reported following the steps of the PRISMA 2020 reporting guideline (Appendix: Supplemental Table S1). The pre-developed protocol was registered in the International Prospective Register of Systematic Reviews (CRD42020148160) and published as a peer-reviewed paper.^53^

### Search strategy and data sources

In collaboration with an information specialist, a comprehensive search strategy was developed, incorporating relevant keywords, MeSH terms from published reviews, and palliative care filters. The search strategy was refined and applied across four bibliometric databases, EMBASE, HTA Database, MEDLINE, and NHS EE Database. The search strategies for all bibliometric databases can be found in Appendix: Supplemental Table S2. The initial search was performed on July 16th, 2019 and updated on June 4th, 2024 considering literature from January 1st, 2010 onwards. Furthermore, reference lists of included publications and existing systematic reviews on economic evidence in palliative and end-of-life care settings were manually searched to identify any relevant articles that may have been missed.

### Screening process

Identified records were imported into the software *Covidence*.^54^ Duplicates were removed using both automated and manual methods. In two rounds of independent screening, two researchers each (ES and MB or CF, respectively) assessed the eligibility of the records. The initial screening was based on the titles and abstracts of the articles, followed by screening the full texts. In case of discrepancies, a third reviewer (EM or JS) was consulted to reach a final decision.

### Inclusion criteria

The inclusion criteria were based on the PICOS framework.

**P**opulation: Palliative (including end-of-life) care patients aged 18 years or older, treated in any setting, such as hospital, home, hospice, nursing home. Palliative care was defined as ‘*an approach that improves the quality of life of patients and their families who are facing problems associated with life-threatening illness. It prevents and relieves suffering through the early identification, impeccable assessment and treatment of pain and other problems, whether physical, psychosocial, or spiritual’.*^55^ End-of-life care was defined as a special form of palliative care provided to individuals in the last weeks or months of their lives.^56^

**I**ntervention & **C**omparator: Any type of interventions with suitable comparator related to palliative and/or end-of-life care.

**O**utcome: Costs, outcomes, and cost-effectiveness.

**S**tudy Design: Full economic evaluations as defined by Drummond et al. ‘*the comparative analysis of alternative courses of action in terms of both their costs and consequences*’.^18^ This includes specific types of analyses distinguished by their outcomes, such as cost-consequence analyses, cost-minimisation analyses, cost-effectiveness analyses, cost-utility analyses, and cost-benefit analyses.^57^ A cost-consequence analysis reports costs and effects separately in a disaggregated manner, allowing decision-makers to consider each element individually. Cost-minimisation analysis is used when interventions are assumed to produce equivalent outcomes, comparing only their costs to identify the least expensive option. Cost-effectiveness analysis compares the costs of interventions relative to a specific health outcome (e.g. life years gained or cases prevented) to determine which option offers the best value for money within a particular disease area. Cost-utility analysis is a type of cost-effectiveness analysis that expresses outcomes in QALYs, enabling comparisons across different health conditions by capturing both the quantity and quality of life. Cost-benefit analysis expresses both costs and outcomes in monetary terms, facilitating comparisons across sectors (e.g. healthcare and education), but it is often criticised for the additional methodological challenges in valuation.^18,58^

The palliative and/or end-of-life care criteria were determined based on the primary aim of the intervention. Trials were included if the primary objective was palliative symptom relief or if the intervention was explicitly stated to be palliative or end-of-life care. Trials with a primary curative intent were excluded from the review. In case of doubt, a palliative care clinician (EM) was consulted. The study design criterion was guided by perceived design due to potential discrepancies between reported and actual designs and required a comparative analysis. There were no geographical restrictions, but only studies published in English or German (determined by reviewers’ proficiency) were considered.

### Data extraction

A customised data extraction form was developed in *Covidence*^54^ and pilot-tested on 10 articles. Revisions were made based on the results of the pilot test. Extracted data are presented in Appendix: Supplemental Table S3. Additionally, online information regarding the required or recommended perspective and cost-effectiveness threshold from national decision-making guidelines (that were effective at the time of the study) were collected. Study authors were contacted to seek clarification of the methods used or to obtain additional data, where necessary. Data extraction was performed by ES and CF.

### Data analysis

The extracted descriptive and methodological data elements were synthesised using a narrative approach, following the recommendations of the Cochrane Consumers and Communication Review Group.^59^

### Quality assessment

Furthermore, included studies were assessed for reporting quality using the up-to-date 28-item Consolidated Health Economic Evaluation Reporting Standards (CHEERS) checklist,^60^ and methodological quality using the Consensus on Health Economic Criteria (CHEC) list.^61^ Two authors were involved in double-assessment (ES, CF or MB) with potential conflicts reviewed by a third assessor (JS). The reporting quality and methodological quality are presented in an aggregated form per checklist item, using graphs. Per CHEERS item, each study could score 1 point if fully met, 0.5 points if partially met, and 0 points if insufficient information was reported. Per CHEC item, each study could score 1 point if fully met, 0.5 points if the criterium was partly fulfilled, and 0 points if the criterium was not fulfilled. For both quality checklists, we generated an overall percentage score, giving all items equal weights (items not applicable for the respective study were excluded from the calculation). We categorised studies as high quality when overall percentage scores were 75% or higher, moderate quality for 50%–74%, and low quality for lower than 50%.^62^

### Role of the funding source

The funder of the study had no involvement in study design, data collection, data analysis, data interpretation, or the writing of the report.

## Results

**Figure 1.**
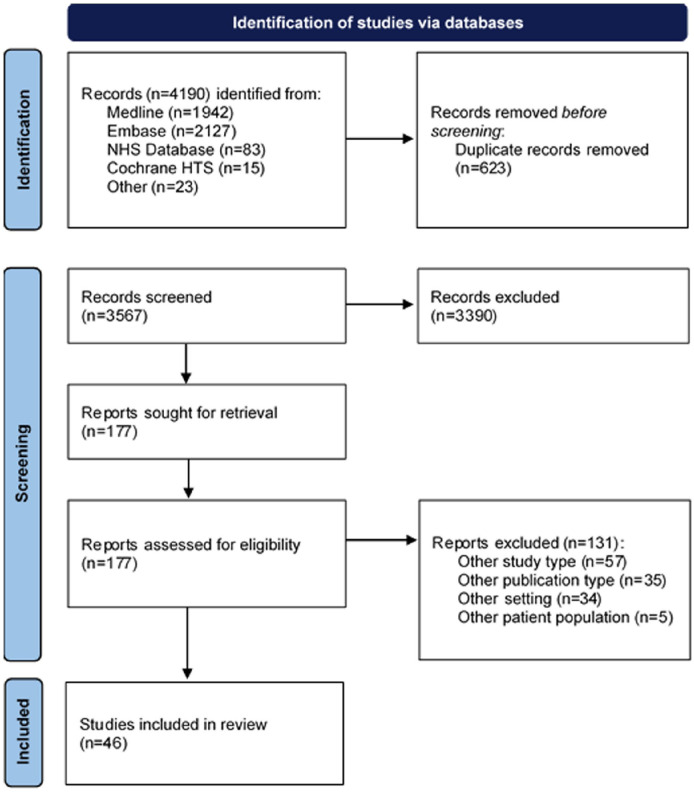
PRISMA flow chart.

Figure 1 presents the study selection process. We identified 4190 references, from which 177 studies underwent full-text review. Overall, 46 studies^63–108^ met the eligibility criteria and were included in the analysis. Nine studies^68–72,93,94,103,105^ employed two types of economic evaluations (e.g. cost-utility and cost-effectiveness analyses) and eight studies^66,90,93,98,100–102,108^ analysed more than one intervention leading to 73 comparisons of costs and effects. Main exclusion reasons at the full-text review stage are presented in Appendix: Supplemental Table S4.

### Main study characteristics

**Figure 2.**
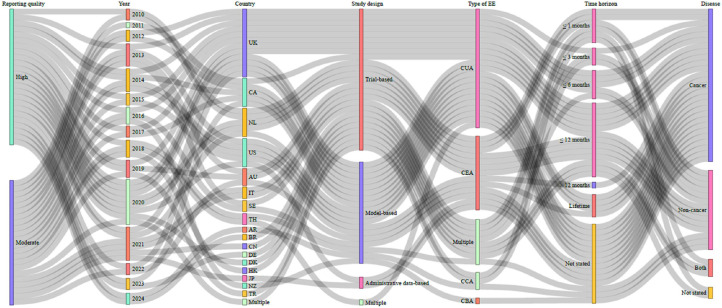
Interrelation between study characteristics across studies. ARG: Argentina; AUS: Australia; BEL: Belgium; BRA: Brazil; CAN: Canada; CHE: Switzerland; DEU: Germany; DNK: Denmark; FIN: Finland; GBR: Great Britain; HKG: Hong Kong; ITA: Italy; JPN; Japan; NLD: Netherlands; NZL: New Zealand; POL: Poland; SWE: Sweden; THA: Thailand; TUR: Turkey; USA: United States of America; EE: economic evaluation; CCA: cost-consequence analysis; CBA: cost-benefit analysis; CEA: cost-effectiveness analysis; CMA: cost-minimisation analysis; CUA: cost-utility analysis.

The main characteristics of the studies are presented in Table 1, while their interrelation is visualised in Figure 2.

**Table 1.** Descriptive characteristics of the included studies.

Author	Year	Journal	Country of data origin	Study type	Disease area	Perceived type of care	Intervention	Comparator	Sample size	Perceived type of economic evaluation	Cost-effective
Bauer et al.^63^	2020	*Health Soc Care Community*	GBR	Model-based	NS	EOLc	ACP intervention	UC	NS	CUA	+
Bennett et al.^64^	2017	*Health Technol Assess*	GBR	Model-based	Advanced diseases (heterogeneous)	PC	Pain self-management	UC	248	CUA	++
Cartoni et al.^65^	2021	*J Palliat Med*	ITA	Trial-based	Cancer: haematologic malignancies	PC; EOLc	Home care programme	UC	119	CEA	+
Chang et al.^66^	2020	*J Vasc Interv Radiol*	USA	Model-based	Cancer: bone metastases	PC	^1)^SFRT & ablation ^2)^Ablation & SFRT ^3)^MFRT & ablation ^4)^Ablation & MFRT	^(1)&(2)^SFRT & SFRT ^(3)&(4)^MFRT & MFRT	NS	^(1)–(4)^CUA	^(1)^+ ^(2)^ **-** ^(3)^+ ^(4)^ **-**
Collinson et al.^67^	2016	*J Med Imaging Radiat Oncol*	NZL	Model-based	Cancer: stage IV metastatic breast, prostate & lung cancers	PC	Single-fraction EBRT	Multiple-fraction EBRT	NS	CEA	++
Earnshaw et al.^68^	2010	*Aliment Pharmacol Ther*	NLD	Model-based	Advanced diseases (heterogeneous)	PC	Medication for opioid-induced constipation	UC	NS	^(1)^CEA ^(2)^CUA	^(1)^+ ^(2)^+
El Alili et al.^69^	2020	BMC *Health Serv Res*	NLD	Trial-based	Advanced dementia	PC	Psychosocial group sessions with family caregivers & people with dementia	UC	231	^(1)^CEA ^(2)^CUA	^(1)^+**|++**^b^ ^(2)^++
El Alili et al.^70^	2020	*Palliat Med*	NLD	Trial-based	Cancer: metastatic colorectal cancer	PC	Treatment targeting psychological distress	UC	349	^(1)^CEA ^(2)^CUA	^1)^+ ^2)^++
Evans et al.^71^	2021	*Int J Nurs Stud*	GBR	Trial-based	Chronic non-cancer conditions	PC	Community-based PC	UC	50	^(1)^CEA ^(2)^CUA	^(1)^ **/** ^(2)^ **/**
Farquhar et al.^72^	2014	BMC *Med*	GBR	Trial-based	Cancer	PC	BIS (immediately)	BIS (after 2-weeks)	67	^(1)^CEA ^2)^CUA	^(1)^++ ^(2)^++
Farquhar et al.^73^	2016	*Trials*	GBR	Trial-based	Advanced non-malignant diseases (heterogeneous)	PC	BIS (immediately)	BIS (after 4 weeks)	87	CUA	**-**
Froggatt et al.^74^	2020	*Health Technol Assess*	GBR	Trial-based	Dementia	PC	Complex dementia intervention	UC	32	CCA	N/A
Furlan et al.^75^	2012	*Neuro Oncol*	CAN	Model-based	Cancer	PC	Surgery + RT	RT	101	CUA	**-**
Gottschalk et al.^76^	2023	*Ann Palliat Med*	DEU	Trial-based	Advanced non-malignant diseases (heterogeneous)	PC	Home-based PC	UC	172	CUA	**--**
Halling et al.^77^	2020	BMC *Palliat Care*	DNK	Trial-based	Cancer: incurable	PC	Home-based PC	UC	340	CUA	**-**
Hashimoto et al.^78^	2021	*J Palliat Med*	JPN	Administrative data-based	Cancer: stage IV	PC	PC	UC	401	CEA	++
Huo et al.^79^	2014	*Am J Manag Care*	USA	Administrative data-based	Cancer: malignant melanoma (stage IV)	EOLc	Hospice care for 4 or more days	Hospice care for 0–3 days	862	CEA	**/**
Iskedjian et al.^80^	2011	*J Pain Symptom Manage*	CAN	Model-based	Cancer	PC	Methylnaltrexone	Laxatives only	100,000	CBA	++
Jeurnink et al.^81^	2010	*J Gastroenterol*	NLD	Trial-based	Cancer: malignant gastric outlet obstruction	PC	Gastrojejunostomy	Duodenal stent placement	39	CEA	**/**
Johnson et al.^82^	2015	BMC *Med*	GBR	Trial-based	Cancer: intra-thoracic malignancy (primary or secondary tumours)	PC	3 sessions breathing technique training	1 session breathing technique training	156	CUA	**--**
Jones et al.^83^	2013	*J Pain Symptom Manage*	GBR	Trial-based	Cancer: active, progressive, recurrent malignant breast or haematological diseases	Unclear	Rehabilitation intervention	UC	41	CUA	+
Kim et al.^84^	2014	*Int J Radiat Oncol Biol Phys*	USA	Model-based	Cancer: painful vertebral bone metastases	PC	SBRT	Single fraction EBRT	NS	CUA	**-**
Lamfre et al.^85^	2024	*Ann Palliat Med*	ARG	Model-based	Cancer	EOLc	Home-based PC service	Home-based UC	278	CEA	++
Ljungman et al.^86^	2013	*World J Surg*	SWE	Model-based	Cancer: malignancy of the exocrine pancreas or ampulla	PC	PC	Curative resection	444	CCA	N/A
Lowery et al.^87^	2013	*Gynaecol Oncol*	USA	Model-based	Cancer: recurrent platinum-resistant ovarian cancer	PC	Early PC intervention	UC	NS	CUA	++
McCaffrey et al.^88^	2013	BMJ *Support Palliat Care*	AUS	Trial-based	Advanced cancer & other non-cancer life limiting advanced illness (heterogeneous)	PC	Home-based PC	UC	32	CEA	**~**
McCaffrey et al.^89^	2019	*Palliat Med*	AUS	Trial-based	Cancer	PC	Subcutaneous ketamine	Placebo	185	CEA	**--**
Meads et al.^90^	2019	*Int J Technol Assess Health Care*	GBR	Model-based	Advanced cancer	EOLc	^1)^PainCheck ^2)^Tackling Cancer Pain Toolkit	UC	NS	^(1)–(2)^CUA	^(1)–(2)^++
O’Halloran et al.^91^	2020	BMC *Nephrol*	GBR	Trial-based	End-stage kidney diseases	Unclear	Immediate ACP	Deferred ACP	36	CCA	N/A
Pattenden et al.^92^	2012	BMJ *Support Palliat Care*	GBR	Trial-based	Heart failure NYA III or IV	PC	Collaborative PC	UC	99	CEA	**~**
Pham et al.^93^	2014	*Ont Health Technol Assess Ser*	CAN	Model-based	Cancer, CHF, COPD (heterogeneous)	PC; EOLc	^1)^PTC In-home ^2)^PTC Inpatient ^3)^PTC Comprehensive ^4)^PCPD 1 ^5)^PCPD 2 ^6)^PCPD 3 ^7)^Multicomponent psychoeducational interventions ^8)^Supportive interventions for informal carers	UC	NS	CEA, CUA	^(1)^++ ^(2)^++ ^(3)^- ^(4)^++ ^(5)^++ ^(6)^+ ^(7)^- ^(8)^-
Rosato et al.^94^	2021	*Mult Scler Relat Disord*	ITA	Trial-based	Severe multiple sclerosis	PC	Home-based PC	UC	76	^(1)^CEA ^(2)^CUA	^(1)^++ ^(2)^/
Sahakyan et al.^95^	2023	*J Clin Oncol*	CAN	Trial-based	Cancer	PC	Geriatric assessment & management plus UC	UC	160	CUA	**--**
Sahlen et al.^96^	2016	*Palliat Med*	SWE	Trial-based	CHF	PC	Integrated palliative advanced homecare	UC	72	CUA	++
Sangmala et al.^97^	2018	*J Med Assoc Thai*	THA	Model-based	Cancer: advanced hepatocellular carcinoma	PC	Sorafenib treatment	PC	180	CUA	**--**
Saygili et al.^98^	2019	*Eur J Cancer Care*	TUR	Trial-based	Cancer	PC	^(1)^Comprehensive PC centre ^(2)^Hospital inpatient services	Home healthcare services	160	^(1)–(2)^CEA	^(1)^-- ^(2)^/
Sellars et al.^99^	2022	*PLoS One*	AUS^a^	Model-based	End-stage kidney diseases	EOLc	ACP intervention	UC	NS	CEA	+
Shafiq et al.^100^	2015	*J Bronchology Interv Pulmonol*	USA	Model-based	Cancer: malignant pleural effusion	PC	^(1)^TS ^(2)^TP ^(3)^TPC ^(4)^RPP	Palliative intervention (repeated thoracentesis)	NS	^(1)–(4)^CUA	^(1)^ **-** ^(2)^ **--** ^(3)^+ ^(4)^ **--**
Suttichai-mongkol et al.^101^	2018	*J Med Assoc Thai*	THA	Model-based; Trial-based	Cancer: unresectable hilar cholangio-carcinoma	PC	^(1)^EBD ^(2)^PTBD	PC	274	^(1)–(2)^CUA	**-**
Thein et al.^102^	2017	*PLoS One*	CAN	Model-based	Cancer: hepatocellular carcinoma	PC	^(1)^TACE or TACE & sorafenib ^(2)^Sorafenib alone ^(3)^Non-sorafenib chemotherapy alone	No treatment or BSC	1172	^(1)–(3)^CUA	^(1)^+ ^(2)^– ^(3)^+
Verberkt et al.^103^	2021	*Respir Med*	NLD	Trial-based	COPD	PC	Oral sustained-release morphine	Placebo	124	^(1)^CEA ^(2)^CUA	^(1)^++ ^(2)^++
Vieira et al.^104^	2024	*Pain Pract*	BRA	Trial-based	Cancer: gastrointestinal neoplasia	PC; EOLc	Epidural morphine & ropivacaine	Oral morphine	24	CEA	**/**
Wichmann et al.^105^	2020	BMC *Med*	BEL, FIN, ITA, NLD, POL, CHE, GBR	Trial-based	NS	PC	PC intervention	UC	551	^(1)^CCA ^(2)^CMA	^(1)^N/A ^(2)^N/A
Wong et al.^106^	2018	*Palliat Med*	HKG	Trial-based	End-stage HF	PC	Home-based PC	UC & placebo social calls	84	CUA	++
Wu et al.^107^	2021	BMC *Palliat Care*	CHN	Trial-based	Cancer: advanced breast, prostate, lung, colorectal & other malignancies	PC	PC	UC	248	CUA	**/**
Yi et al.^108^	2022	*Thorax*	GBR	Model-based	COPD, lung cancer, interstitial lung disease	PC	^(1)^Breathlessness service + UC ^(2)^Breathlessness service with lasting effects + UC	UC	1000	CUA	++

Superscript numbers (1) to (8) indicate when more than one intervention or types of economic evaluations were conducted. Results for Pham et al.^93^ are for the cost-utility analysis.

Country: ARG: Argentina; AUS: Australia; BEL: Belgium; BRA: Brazil; CAN: Canada; CHE: Switzerland; DEU: Germany; DNK: Denmark; FIN: Finland; GBR: Great Britain; HKG: Hong Kong; ITA: Italy; JPN: Japan; NLD: Netherlands; NZL: New Zealand; POL: Poland; SWE: Sweden; THA: Thailand; TUR: Turkey; USA: United States of America.

Disease and type of care: COPD: chronic obstructive pulmonary disease; CHF: chronic heart failure; EOLc: end-of-life care; PC: palliative care.

Intervention/comparator: ACP: advance care planning; BIS: breathlessness intervention service; BSC: best supportive care; EBD: endoscopic biliary drainage; EBRT: external beam radiotherapy; EOL: end-of-life; MFRT: multiple-fraction radiation therapy; PC: palliative care; PCPD: patient care planning discussions; PTBD: percutaneous transhepaticbiliary drainage; PTC: palliative team care; RPP: rapid pleurodesis protocol; RT: radiation therapy; SBRT: stereotactic body radiation therapy; SFRT: single-fraction radiation therapy; TACE: transarterial chemoembolization; TP: thoracoscopic talc poudrage; TPC: tunnelled pleural catheter; TS: chest tube-guided talc slurry; UC: usual care.

Type of economic evaluation: CCA: cost-consequence analysis; CBA: cost-benefit analysis; CEA: cost-effectiveness analysis; CMA: cost-minimisation analysis; CUA: cost-utility analysis.

Cost-effectiveness results: ++ = dominant or positive result (for cost-benefit analysis); + = cost-effective; ~ = inconclusive (because of the results); - = not cost-effective; -- = dominated; / = unclear (because of incomplete reporting); N/A = not applicable; NS = not stated.

a The author of the study was contacted to clarify the data origin.

b In this study, two different measures were used for the cost-effectiveness analysis and results differed.

Out of the 46 studies, 50% were published in the last 5 years (*n* = 23).^63,65,66,69–71,74,76–78,85,89–91,94,95,98,99,103–105,107,108^ In total, 87% (*n* = 40)^63–84,86–96,99,100,102,103,105,106,108^ of the included studies investigated data from high-income countries (Australia, Belgium, Canada, Denmark, Germany, Hong Kong, Italy, Japan, Netherlands, New Zealand, Sweden, United Kingdom, United States of America, Finland, Poland, Switzerland) with the remaining 13% (*n* = 6)^85,97,98,101, 104,107^ from upper-middle income countries (Argentina, Brazil, China, Thailand, Turkey). All studies,^63–108^ except one,^105^ were based on single-country data, most commonly from the United Kingdom (*n* = 12, 26%).^63,64,71–74,82,83,90–92,108^

Over 50% of the studies (*n* = 25)^65,69–74,76,77,81–83,88,89,91,92,94–96,98,103–107^ were trial-based, 39% (*n* = 18)^63^,^64^,^66^–68,^75,80^,^84–87,90,93,97,99,100,102,108^ were model-based, one study used both trial- and model-based approaches,^101^ while the others were based on administrative data.^78,79^ Some studies were not limited to one disease area but included heterogeneous diseases (*n* = 8, 17%).^64,68,71,73,76,88,93,108^ Single disease studies focussed on cancer (*n* = 27, 59%),^65–67,70,72,75,77–87,89,90,95,97,98,100–102,104,107^ heart failure (*n* = 3, 7%),^92,96,106^ dementia (*n* = 2, 4%),^69,74^ kidney diseases (*n* = 2, 4%),^91,99^ COPD (*n* = 1, 2%),^103^ and multiple sclerosis (*n* = 1, 2%).^94^ The majority of the studies (*n* = 36, 78%)^64,66–78,80,82,84,86–89,92,94–98,100–103,105–108^ were conducted in the palliative care setting. The interventions evaluated ranged from broad, undefined palliative care to very specific symptom management treatments, for example for pain. The main comparator was usual care (*n* = 26, 57%).^63–65,68–71,74,76–78,83,87,88,90,92–96,99,105–108^ Of the 46 studies, 46% conducted a cost-utility (*n* = 21)^63,64,66,73,75–77,82–84,87,90,95–97,100–102,106–108^ and 26% a cost-effectiveness analysis (*n* = 12),^65,67,78,79,81,85,88,89,92,93,98,99,104^^[Bibr bibr104-02692163261418546]^ 8 studies (17%)^68–72,93,94,103^ both, cost-effectiveness and cost-utility analyses, 3 studies (7%)^74,86,91^ a cost-consequence analysis, 1 study (2%)^80^ a cost-benefit analysis and 1 study (2%)^105^ both, a cost-minimisation and cost-consequence analysis. Sample sizes of trial-based economic evaluations varied between 24 and 551 (mean of 146), with 22 studies (85%)^65,71–76,81–83,88,89,91,92,94–98,103,104,106^ having fewer than 200 participants.

### Methodological study characteristics

Table 2 gives an overview of further methodological study characteristics.

**Table 2.** Methodological characteristics of the included studies.

		Context											Outcome measurement								Costing																
		Setting(s)					Time horizon	Type(s) of economic evaluation					For whom?		Utility measurement tool	Other outcome measures	Data collection methods				Costs included						Sources of resource use data				Sources of unit cost data						
Author	Year	Hospital	Home setting	Nursing homes	Hospice	Other		CUA	CEA	CBA	CCA	CMA	Patient	Informal caregiver			Self-reported	Assisted	Proxy	Not stated	Healthcare	Social care	Patient (direct)	Family (direct)	Informal care	Productivity loss	Administrative data	Patients’ self-report	Clinician-report	Literature	Unit cost database	Administrative data	Published studies/reports	Tariffs	Expert opinion	Study-specific calculation	Unspecified source
Bauer et al.^63^	2020	x	x	x	x		⩽12 M	x						x	EQ-5D			N/A			x	x								x	x		x		x		
Bennett et al.^64^	2017	x			x		⩽12 M	x					x		EQ-5D-3L		x				x	x						x			x	x		x			
Cartoni et al.^65^	2021	x	x				⩽1 M		x				x			Days of care prevented	x				x		x	x			x							x		x	x
Chang et al.^66^	2020	x					Lifetime	x					x		NS			N/A			x						x					x		x			
Collinson et al.^67^	2016	x					Lifetime		x				x			Disability-weighted life years		N/A			x		x				x				x		x			x	
Earnshaw et al.^68^	2010			NS			⩽12 M	x	x				x		EQ-5D	Time without constipation		N/A			x									x		x	x		x		
El Alili et al.^69^	2020			x			⩽12 M	x	x				x	x	EQ-5D-3L	GAIN; QUALID			x		x	x		x		x	x	x			x			x			
El Alili et al.^70^	2020	x					⩽12 M	x	x				x		EQ-5D-5L	HADS	x				x		x		x	x		x	x		x		x	x		x	
Evans et al.^71^	2021					x	⩽3 M	x	x				x		EQ-5D-5L	IPOS score	x		x		x	x	x	x	x		x	x			x						
Farquhar et al.^72^	2014		x				NS	x	x				x	x	EQ-5D	NRS distress due to breathlessness	x				x	x		x	x			x			x			x			
Farquhar et al.^73^	2016		x				NS	x					x	x	EQ-5D		x				x	x						x			x			x			
Froggatt et al.^74^	2020			x			⩽ 1 M				x		x			EQ-5D-5L; ICECAP-SCM; ICECAP-O			x		x	x					x	x			x	x					
Furlan et al.^75^	2012	x					NS	x					x		NS			N/A			x									x	x	x	x				
Gottschalk et al.^76^	2023		x			x	⩽12 M	x					x		EQ-5D-5L		x		x		x		x	x	x			x	x		x			x			
Halling et al.^77^	2020	x	x				⩽6 M	x					x	x	EORTC QLQ-C30; SF-6D					x	x	x	x	x	x		x	x		x		x	x	x			
Hashimoto et al.^78^	2021	x					NS		x				x			NRS pain scale		x			x						x							x			
Huo et al.^79^	2014				x		⩽3 M		x				x					N/A			x						x					x					
Iskedjian et al.^80^	2011			NS			NS			x			N/A					N/A			x									x				x			
Jeurnink et al.^81^	2010	x					NS		x				x			Functional GOOSS score			x		x						x					x					
Johnson et al.^82^	2015	x	x		x		NS	x					x		EQ-5D					x	x								NS								x
Jones et al.^83^	2013				x		⩽3 M	x					x		EQ-5D		x				x							x			x						
Kim et al.^84^	2014	x					Lifetime	x					x		NS			N/A			x						x					x					
Lamfre et al.^85^	2024	x	x				⩽12 M		x				x			Days at home; place of death		N/A			x				x					x			x	x			
Ljungman et al.^86^	2013	x					NS				x		x			SF-6D		N/A			x						x					x		x			
Lowery et al.^87^	2013	x					⩽6 M	x					N/A		NS			N/A			x						x			x		x	x				
McCaffrey et al.^88^	2013		x				⩽1 M		x				x			Days at home				x	x									x	x	x		x			
McCaffrey et al.^89^	2019	x					⩽1 M		x				x			FACIT-Pal HRQOL				x	x						x	x			x			x			
Meads et al.^90^	2019		x				⩽12 M	x					x		EQ-5D-3L					x	x	x								x	x			x			
O’Halloran et al.^91^	2020	x					⩽3 M				x		x			SF-6D; IST 15; CORE 34; SHARED		x			x	x						x			x			x			
Pattenden et al.^92^	2012		x			x	NS		x				x			Hospital admission averted				x	x						x				x	x					
Pham et al.^93^	2014	x	x				⩽12 M	x	x				x	x	EQ-5D	Days at home; % dying at home		N/A			x						x			x		x	x		x		
Rosato et al.^94^	2021	x					⩽6 M	x	x				x		EQ-5D-3L	POS-S-MS	x				x		x					x			x			x			
Sahakyan et al.^95^	2023					x	⩽12 M	x					x		EQ-5D-5L		x	x			x		x	x	x	x	x	x	x		x	x		x			
Sahlen et al.^96^	2016	x					⩽6 M	x					x		EQ-5D					x	x						x					x		x			
Sangmala et al.^97^	2018	x					Lifetime	x					x		CLDQ			N/A			x						x						x	x			
Saygili et al.^98^	2019	x	x				NS		x				x			EORTC QLQ-C30		x			x		x	x			x	x									x
Sellars et al.^99^	2022	x					⩽12 M		x				x			End-of-life preferences being met		N/A			x						x						x				
Shafiq et al.^100^	2015	x					⩽6 M	x					x		EQ-5D			N/A			x						x					x	x		x	x	
Suttichaimongkol et al.^101^	2018	x					NS	x					x		EQ-5D-3L					x	x		x				x					x					
Thein et al.^102^	2017	x	x				NS	x					x		NS			N/A			x						x				x	x					
Verberkt et al.^103^	2021		x				⩽1 M	x	x				x		EQ-5D-5L	CAT score	x				x		x	x	x	x		x			x	x		x			x
Vieira et al.^104^	2024	x					⩽12 M		x				x			Pain reduction				x	x						x		x			x		x			
Wichmann et al.^105^	2020			x			⩽1 M				x	x	x			EQ-5D-5L; QOD-LTC			x		x						x	x			x	x					
Wong et al.^106^	2018		x				NS	x					x		SF-6D					x	x						x					x		x			
Wu et al.^107^	2021	x					NS	x					x		CQLQ					x	x						x					x					
Yi et al.^108^	2022	x	x			x	>12 M	x					x		EQ-5D			N/A			x	x								x	x	x					

N/A = not applicable; NS = not stated; M = months; *Instruments*: CAT: COPD assessment test (HRQoL); CORE 34: clinical outcomes in routine evaluation; CLDQ: chronic liver disease questionnaire; CQLQ: Chinese quality of life questionnaire; EORTC QLQ-C30: EORTC core quality of life of cancer patients; EQ-5D: EuroQol – 5 dimensions – 5 levels or 3 levels; FACIT-Pal: functional assessment of chronic illness therapy-palliative care (HRQoL); GAIN: gain in alzheimer care instrument; GOOSS: gastric outlet obstruction scoring system; HADS: hospital anxiety and depression scale; ICECAP-O: ICECAP older adults; ICECAP-SCM: ICECAP supportive care measure; IPOS: integrated palliative care outcome scale; IST15: Isaacs set test (degree of cognitive impairment); NRS: numerical rating scale; POS-S-MS: palliative care outcome scale – symptoms – multiple sclerosis; QOD-LTC: quality of dying – long term care; QUALID: quality of life in late-stage dementia; SF-6D: short form – 6 dimensions; SHARED: patient experience of shared decision making.

#### Setting, perspective, time horizon and discounting

Nineteen studies (41%)^66,67,70,75,78,81,84,86,87,89,91,94,96,97,99–101,104,107^ were conducted in the hospital setting, 12 (26%)^63–65,76,77,82,85,92,93,98,102,108^ in more than one setting (e.g. hospital and home care), six (13%)^72,73,88,90,103,106^ in the home care setting, while only seven (15%) were conducted in nursing homes,^69,74,105^ hospices,^79,83^ or primary/cancer care centres.^71,95^ Two studies^68,80^ did not report the setting. In total, 35 studies (76%)^63–70,74,77,79–81,84–86,88–90,93–98,100–106,108^ reported their analytical perspective. Of these, all but one, which stated a societal perspective but was perceived as a healthcare perspective,^81^ reflected the perceived perspective. As shown in Table 3, 58% of the perceived perspectives were in accordance with the country-specific decision-making guidelines.

**Table 3.** Decision-making characteristics and quality of the included studies.

Author	Year	Country of study origin	Analytical perspective		CE decision threshold			
			Perceived	Required/ recommended by methods guidelines	National CE decision threshold	Applied CE decision threshold	CHEERS %	CHEC %
Bauer et al.^63^	2020	GBR	Health and social care	Health and social care	£20,000–£30,000/QALY	£20,000, £30,000/QALY	79%	94%
Bennett et al.^64^	2017	GBR	Health and social care	Health and social care	£20,000–£30,000/QALY	£20,000	90%	92%
Cartoni et al.^65^	2021	ITA	Provider	National health service	Not specified	NS	60%	76%
Chang et al.^66^	2020	USA	Payer	Payer	US$50,000–100,000/QALY	US$100,000/QALY	78%	74%
Collinson et al.^67^	2016	NZL	Healthcare	Payer	Not specified	NZ$45,000/disability-weighted LY	77%	79%
Earnshaw et al.^68^	2010	NLD	Payer	Societal	Not specified	€50,000/QALY	79%	86%
El Alili et al.^69^	2020	NLD	Societal	Societal	Not specified	€0, €10,000, €20,000/QALY	78%	94%
El Alili et al.^70^	2020	NLD	Societal	Societal	Not specified	€0, €20,000, €80,000/QALY	83%	94%
Evans et al.^71^	2021	GBR	Societal	Health and social care	£20,000–£30,000/QALY	NS	73%	83%
Farquhar et al.^72^	2014	GBR	Societal	Health and social care	£20,000–£30,000/QALY	NS	63%	74%
Farquhar et al.^73^	2016	GBR	Health and social care	Health and social care	£20,000–£30,000/QALY	£20,000–£30,000/QALY	63%	68%
Froggatt et al.^74^	2020	GBR	Health and social care	Health and social care	£20,000–£30,000/QALY	NS	81%	82%
Furlan et al.^75^	2012	CAN	Payer	Public healthcare payer	Not specified, but C$50,000/QALY often cited	US$50,000/QALY	63%	63%
Gottschalk et al.^76^	2023	DEU	Societal	Healthcare	Variable	€0–€120,000/QALY	96%	86%
Halling et al.^77^	2020	DNK	Societal	Societal	Not specified	€80,000/QALY	91%	89%
Hashimoto et al.^78^	2021	JPN	Provider (hospital)	Public healthcare payer	US$50,000 = ¥5 million/QALY	US$50,000 = ¥5 million/QALY	75%	61%
Huo et al.^79^	2014	USA	Payer	Payer	US$50,000–100,000/QALY	NS	63%	72%
Iskedjian et al.^80^	2011	CAN	Payer	Public healthcare payer	Not specified, but C$50,000/QALY often cited	N/A	65%	79%
Jeurnink et al.^81^	2010	NLD	Healthcare	Societal	Not specified	NS	54%	74%
Johnson et al.^82^	2015	GBR	Healthcare	Health and social care	£20,000–£30,000/QALY	£20,000/QALY	59%	76%
Jones et al.^83^	2013	GBR	Healthcare	Health and social care	£20,000–£30,000/QALY	£20,000, £30,000/QALY	58%	75%
Kim et al.^84^	2014	USA	Payer	Payer	US$50,000–100,000/QALY	US$100,000/QALY	80%	79%
Lamfre et al.^85^	2024	ARG	Health and informal care	Public healthcare payer or societal	Not specified	NS	82%	91%
Ljungman et al.^86^	2013	SWE	Healthcare	Societal	Not specified	N/A	65%	74%
Lowery et al.^87^	2013	USA	Healthcare	Payer	US$50,000–100,000/QALY	$50,000; $100,000/QALY	61%	79%
McCaffrey et al.^88^	2013	AUS	Healthcare	Healthcare system	AU$50,000/QALY	NS	78%	74%
McCaffrey et al.^89^	2019	AUS	Healthcare	Healthcare system	AU$50,000/QALY	NS	81%	89%
Meads et al.^90^	2019	GBR	Health and social care	Health and social care	£20,000–£30,000/QALY	£20,000/QALY	94%	89%
O’Halloran et al.^91^	2020	GBR	Health and social care	Health and social care	£20,000–£30,000/QALY	N/A	69%	81%
Pattenden et al.^92^	2012	GBR	Healthcare	Health and social care	£20,000–−£30,000/QALY	NS	67%	56%
Pham et al.^93^	2014	CAN	Healthcare	Public healthcare payer	Not specified, but C$50,000/QALY often cited	C$50,000/QALY	83%	83%
Rosato et al.^94^	2021	ITA	Healthcare & patient	National health service	Not specified	NS	79%	92%
Sahakyan et al.^95^	2023	CAN	Societal	Public healthcare payer	Not specified, but C$50,000/QALY often cited	C$50,000/QALY	92%	92%
Sahlen et al.^96^	2016	SWE	Healthcare	Societal	Not specified	NS	67%	75%
Sangmala et al.^97^	2018	THA	Payer	Societal	THB 160,000/QALY	THB 160,000/QALY	78%	83%
Saygili et al.^98^	2019	TUR	Healthcare & patient/family	Provider and payer	Not specified	NS	63%	65%
Sellars et al.^99^	2022	AUS	Healthcare	Healthcare system	AU$50,000/QALY	AU$50,000/adhered treatment preferences	65%	75%
Shafiq et al.^100^	2015	USA	Payer	Payer	US$50,000–100,000/QALY	US$100,000/QALY	78%	85%
Suttichaimongkol et al.^101^	2018	THA	Healthcare	Societal	THB 160,000/QALY	THB 160,000/QALY	74%	76%
Thein et al.^102^	2017	CAN	Healthcare	Public healthcare payer	Not specified, but C$50,000/QALY often cited	C$0–100,000/QALY	67%	81%
Verberkt et al.^103^	2021	NLD	Societal	Societal	Not specified	€20,000–€80,000/QALY	96%	92%
Vieira et al.^104^	2024	BRA	Healthcare	Public healthcare payer or societal	Not specified	NS	81%	79%
Wichmann et al.^105^	2020	Multiple	Healthcare	Healthcare or societal perspective	Not specified	€20,000–80,000/QALY	88%	69%
Wong et al.^106^	2018	HKG	Healthcare	Not specified	Not specified	HK$200,000, HK$328,117/QALY	71%	82%
Wu et al.^107^	2021	CHN	Healthcare (patient)	Societal	1–3 times national GDP per capita/QALY	NS	52%	68%
Yi et al.^108^	2022	GBR	Health and social care	Health and social care	£20,000–£30,000/QALY	NS	90%	79%

ARG: Argentina; AUS: Australia; BEL: Belgium; BRA: Brazil; CAN: Canada; CHE: Switzerland; DEU: Germany; DNK: Denmark; FIN: Finland; GBR: Great Britain; HKG: Hong Kong; ITA: Italy; JPN: Japan; NLD: Netherlands; NZL: New Zealand; POL: Poland; SWE: Sweden; THA: Thailand; TUR; Turkey; USA: United States of America; CE: cost-effectiveness; QALY: quality-adjusted life year; CHEERS: consolidated health economic evaluation reporting standards; CHEC: consensus on health economic criteria; NS = not stated; N/A = not applicable.

When reported (32 studies, 70%),^63–71,74,76,77,79,83–85,87–91,93–97,99,100,103–105,108^ the time horizon was 1 month or less in 6 studies (19%)^65,74,88,89,103,105^ and between 1 and 12 months in 21 studies (67%).^63,64,68–71,76,77,79,83,85,87,90,91,93–96,99,100,104^ Where applicable, the reporting and use of discount rates varied (see Appendix: Supplemental Table S5).

#### Outcome methods

Most studies considered only patient outcomes (*n* = 38, 83%).^64–68,70,71,74–76,78,79,81–86,88–92,94–108^ One study^63^ focussed solely on informal caregivers’ outcomes, and others^69,72,73,77,93^ included outcomes for informal caregivers in addition (*n* = 5, 11%). For cost-utility analyses (*n* = 29),^63,64,66,68–73,75–77,82–84,87,90,93–97,100–103,106–108^ QALYs were predominantly based on generic instruments (*n* = 24, 83%),^63,64,68–73,76,82,83,90,93–97,100,101,103,106–108^ like EQ-5D or SF-6D (see Table 2, Supplemental Table S5). For the other types of analyses (*n* = 23),^65,67–72,74,78,81,85,86,88,89,91–94,98,99,103–105^ outcomes were heterogeneous including different patient-reported outcome scores (*n* = 11, 48%),^69–71,74,86,89,91,94,98,103,105^ clinical outcomes (*n* = 5, 22%),^68,72,78,81,104^ and others such as days at home or place of death (*n* = 7, 30%).^65,67,85,88,92,93,99^ Primary data collection (*n* = 29) was either self-reported (*n* = 8, 28%),^64,65,70,72,73,83,94,103^ proxy reported (*n* = 4, 14%),^69,74,81,105^ assisted (*n* = 3, 10%),^78,91,98^ mixed (*n* = 3, 10%),^71,76,95^ or not reported (*n* = 11, 38%).^77,82,88–90,92,96,101,104,106,107^ As presented in Appendix: Supplemental Table S5, the frequency of data collection ranged from one time to six times, and data collection intervals varied widely, ranging from more than once a day to 13 months between two assessments.

#### Costing methods

In total, 54% of the studies (*n* = 25)^66,68,75,78–84,86–89,92,93,96,97,99,100,102,104–107^ did not consider costs beyond healthcare. Others included social care costs (*n* = 11),^63,64,69,71–74,77,90,91,108^ direct patient (*n* = 11)^65,67,70,71,76,77,94,95,98,101,103^ or family costs (*n* = 9),^65,69,71,72,76,77,95,98,103^ informal care costs (*n* = 8),^70–72,76,77,85,95,103^ or costs related to employment and productivity loss (*n* = 4).^69^,^70,95,103^ Resource use was mostly based on one source (*n* = 32, 70%)^63–68,72,73,75,78–81,83–86,88,90–92,94,96,97,99–103,106–108^ such as administrative data (*n* = 28),^65–67,69,71,74,77–79,81,84,86,87,89,92,93,95–102,104–107^ patient self-report (*n* = 17),^64,69–74,76,77,83,89,91,94,95,98,103,105^ clinician report (*n* = 4)^70,76,95,104^ or the literature (*n* = 11).^63,68,75,77,80,85,87,88,90,93,108^ Unit costs were derived from administrative data (*n* = 25, 54%),^64,66,68,74,75,77,79,81,84,86–88,92,93,95,96,100–108^ publicly available tariffs (*n* = 24, 52%; e.g. pharmacy retail prices),^64–66,69,70,72,73,76–78,80,85,86,88–91,94–97,103,104,106^ unit cost databases (*n* = 23, 50%),^63,64,67,69–76,83,88–92,94,95,102,103,105,108^ or published studies and reports (*n* = 12, 26%).^63,67,68,70,75,77,85,87,93,97,99,100^

#### Cost-effectiveness results

Of the 73 individual comparisons, 23 (32%)^64,67,69,70,72,78,80,85,90,93,94,96,103,106,108^ showed dominance, 14 (19%)^63,65,66,68–70,83,93,99,100,102^ showed cost-effective results, 13 (18%)^66,73,75,77,84,93,100–102^ showed not cost-effective results, 8 (11%)^76,82,89,95,97,98,100^ showed the intervention being dominated, 2 (3%)^88,92^ had uncertain results, 8 (11%)^71,79,81,94,98,104,107^ had incomplete reporting of cost-effectiveness results, while for five (7%)^74,86,91,105^ such results were not applicable (e.g. cost-consequence analyses). Information about the applied thresholds can be found in Table 3. In 21 studies (46%)^65,67–70,75,77,80,81,85,86,93–96,98,102–106^ and their countries of data origin (*n* = 10), there was no national cost-effectiveness decision threshold specified at the time of the study conduct.

### Quality of reporting

**Figure 3.**
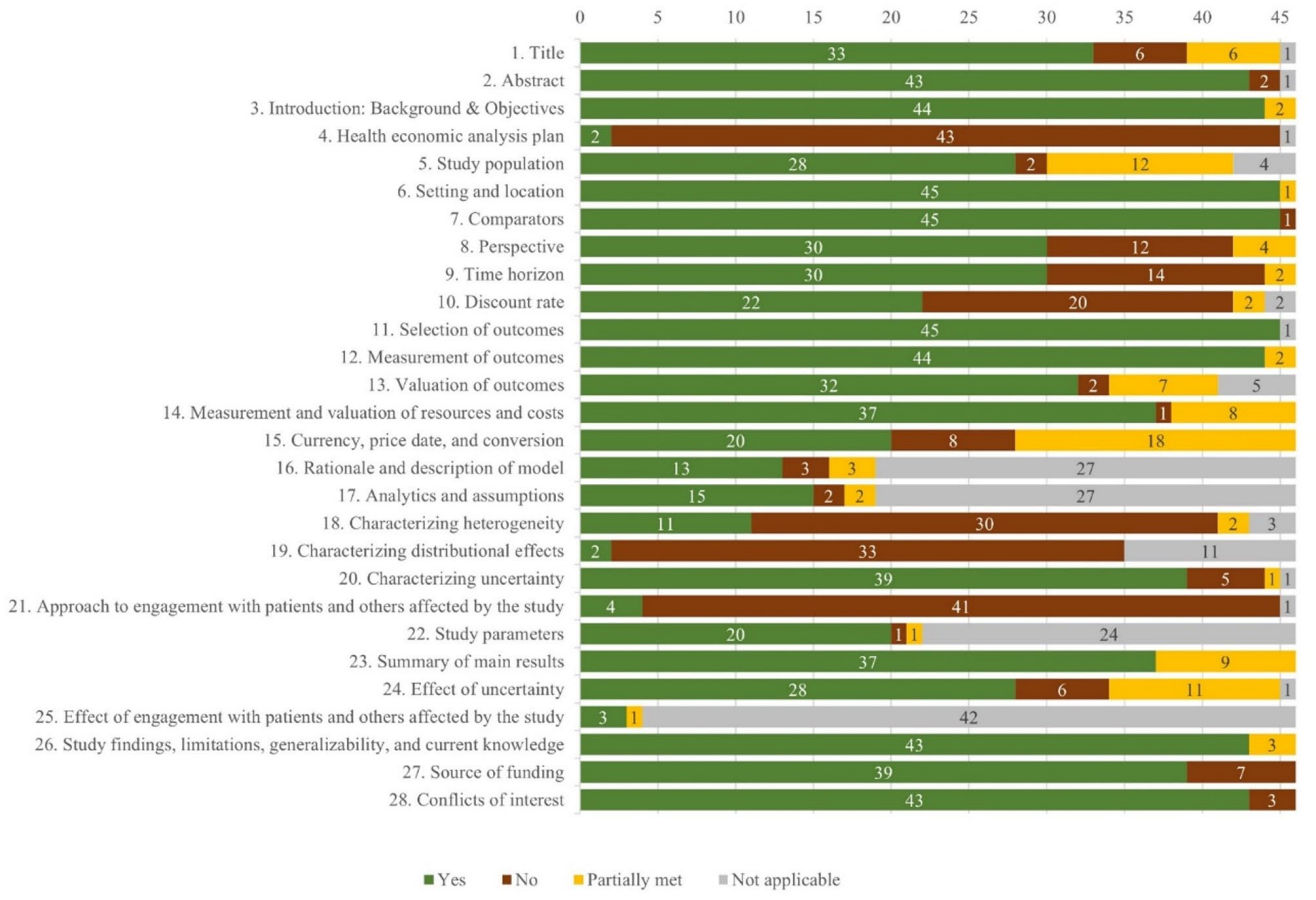
Assessment of reporting quality in included studies using the CHEERS checklist.

The mean quality of reporting was moderate (74%, range: 52%–96%). Figure 3 provides an overview of the CHEERS checklist per item. Most crucially, only 3 studies (7%) had a health economic analysis plan, 13 studies (28%) reported about distributional effects, and 4 studies (9%) reported about Patient and Public Involvement (PPI). The quality of reporting improved with time (Figure 2).

### Quality of conduct

The mean quality of conduct was 80% (range: 56%–94%). Only 18 studies (39%) provided a well-defined research aim or question. The discounting of future costs and outcomes was not applicable in 25 studies (54%) due to the short time horizons. Only three studies (7%) discussed ethical and distributional issues sufficiently (Supplemental Figure S1).

As shown in Table 3, the agreement between the overall CHEERS and CHEC assessment scores was within a 5%-range for 14 studies (30%) and within a 10%-range for 12 studies (26%).

## Discussion

### Main findings of the study

Our systematic review of 46 economic evaluations comprised of 73 comparisons for 65 interventions in the palliative and end-of-life care settings. Most studies were trial-based evaluations of hospital- or home-based interventions in high-income countries. These interventions targeted mainly patients with palliative care needs and were indicated as cost-effective in half of the cases. However, our analysis of the studies’ methodological characteristics revealed that methodological issues previously identified by Fischer and colleagues^21^ are not consistently addressed in practice, which challenges the validity and reliability of the results. We found that the problems were related to variability in reporting and study quality, omission of important costs, small sample sizes and lack of information on missing data, failure to include effects and costs for informal caregivers, poor definition of comparators, and the limited application of a societal perspective, which are likely related to country-specific decision-making guideline requirements.

Our detailed data extraction revealed in how far authors addressed additional methodological challenges and existing recommendations specific to economic evaluations in this area, which have not been explored in previous systematic reviews.

*Patient identification*: Economic evaluation of interventions specifically targeting patients in the end-of-life phase are rare (11%). Due to the lack of a reliable method for determining the onset of end-of-life, there was notable heterogeneity in how the target population was defined in prospective studies. In our systematic review, most studies retrospectively identified the end-of-life patient population by using registry and/or insurance data.

*Generalisability*: No studies from low-middle- or low-income countries were identified. Due to limited generalisability and poor geographical transferability of evidence, mainly because of differing healthcare structures,^109^ there is need for economic evaluations conducted in lower income settings.^2,13^ The provision of palliative care services remains highly heterogeneous across regions, largely due to differences in health policy, resource availability and cultural attitudes towards palliative and end-of-life cares.^110^ While some countries have well-established, publicly funded palliative care systems, others rely heavily on private care or have limited formal infrastructure.^111^ This diversity makes it difficult to draw global conclusions from economic evaluations of palliative and end-of-life care, highlighting the need for tailored economic evaluations that take into account the specific policy and healthcare landscape of each setting, rather than relying on one-size-fits-all recommendations.^111^

*Costing perspective*: The recommendation to adopt a societal perspective is still rarely followed in practice. Adopting a societal perspective that accounts for both healthcare costs and out-of-pocket expenses would also help to uncover the inequalities that may arise in access to care.^13^ However, it should be noted that many decision-making guidelines recommend a narrower perspective than the societal one.

*Comparators*: In most studies, the comparator was usual care but it was often poorly defined, making it unclear how the tested interventions differed from typical care.

*Time horizon*: Most studies had a short time horizon consistent with the context. Four modelling studies^66,67,84,97^ applied a lifetime time horizon (radiotherapy, cancer).

*Outcome selection*: Effects on family members and informal caregivers were mostly omitted likely leading to major missed effects and cost implications of the interventions being tested.^112^ Given the ambiguity in the selection of outcomes to test the effect of palliative and end-of-life care interventions, it is also recommended to test different generic measures alongside disease- or context-specific measures.^113^ Nevertheless, we found only one study that used the ICECAP-SCM in addition to the EQ-5D-5L.^28^

*Outcome measurement*: Data collection in the context of palliative and end-of-life care is considered to be particularly difficult due to the condition of the patient and the need of proxy respondents in many cases. This was also confirmed in our analysis. In most cases, missing data were not reported and when reported, ranged from 3% to 55%.

*Measurement and valuation of costs*: Multiple self-reported resource use questionnaires were applied reflecting that currently there is no consensus on which tool to use. Regarding unit costs, sources varied greatly, although, this is not a unique attribute of the setting.

*Quality of reporting*: We encountered some challenges while extracting data as reporting was inconsistent and often inconclusive. In particular, we had difficulty extracting information on the cost-effectiveness of the interventions, since the necessary data, such as confidence intervals or cost-effectiveness thresholds for decision-making, were often not reported. A further challenge was the inconsistent or incorrect use of terminology across studies concerning the stated type of economic evaluation. In some studies, there was also a mismatch between the reported study perspectives and the actual costs and outcomes included. Only nine studies (20%) adhered to a reporting guideline specific to economic evaluations such as CHEERS,^60^ all of them published in the past 5 years.

*Quality of conduct*: While many studies chose well-suited study designs and identified costs and outcomes appropriately to their chosen perspective, there are opportunities for improvement in defining research questions more clearly, conducting sensitivity analyses, and addressing generalisability.

*Decision-making guidelines*: Only a few national guidelines recommend adopting a societal perspective, which was reflected in the perspectives taken in the economic evaluations of this review. In cases where a societal perspective was recommended for the specific study context (*n* = 11), adherence was seen in four cases (36%). Given that overall adherence to national decision-making guidelines was 58%, adapting them to the specific characteristics of palliative and end-of-life care contexts could significantly enhance the rigour and usefulness of the evidence base.

### What this study adds

Comparing our results with previous systematic reviews shows that the lack of robust health economic evidence base continues to persist, which makes it difficult to accurately evaluate palliative and end-of-life care interventions. Previous systematic reviews in this field highlighted the lack of formal cost-effectiveness studies.^19^ In a systematic review published between 2000 and 2011, Smith and colleagues^23^ identified only one study that was a full economic evaluation, measuring patient outcomes with Palliative Care Outcome Scale (POS-8) and caregiver burden with the Zarit Carer burden inventory.^114^ Overall, 58 out of the 73 comparisons included in the 46 studies allowed determining cost-effectiveness, and half showed dominance or cost-effectiveness. This adds to previous research that palliative care in some instances may be cost-saving,^22,23^ especially in the home care setting.^115,116^ However, these findings should be interpreted with caution due to the limited quality of the evidence. In addition, most studies did not adopt a societal perspective and therefore lack relevant spillover costs and consequences for informal carers.^112,117^ The wide methodological variability of the studies, especially concerning outcome measurement, hindered meaningful comparison across studies. This issue has already been highlighted in earlier publications as well.^118,119^ For instance, a systematic review by Langton et al.^3^ concluded that palliative care reduced resource utilisation and costs in cancer care, yet, the outcomes measured were highly heterogeneous. Hundreds of different metrics were used to evaluate the quality of end-of-life care, none of which provided direct insights into patient or caregiver perceptions. Further reviews focussed on hospital inpatient palliative consultations,^120^ home palliative care services,^121^ all showing inconclusive evidence due to heterogeneity. In more recent reviews, study results were limited by small sample sizes and short durations.^116^ Specific to the oncology setting, few studies considered societal perspectives and the impact on quality of life, which is crucial given the modest survival benefits and significant toxicity associated with many therapies.^122^

In line with the previous systematic literature review on methodological challenges,^21^ key priorities for future analyses include clearly defining the research question and comparator, applying context-specific outcome measures that capture the broad effect of palliative and end-of-life care, conducting sensitivity analyses to improve the assessment of how robust the findings are, and adhering to established economic evaluation reporting standards for more transparency. Regarding the revision of decision-making guidelines, our findings highlight the importance of incorporating a broader, societal perspective and accounting for potential spillover effects that arise within this specific care context.

### Strengths and weaknesses of the study

Our systematic review has both major strengths and weaknesses. Most importantly, our study covers the most up-to-date and comprehensive synthesis of the palliative and end-of-life care economic evaluation literature trying to address the impacts of the increasingly changing landscape of the given care settings, decision-making regulations and some methodological developments on the quantity and quality of the available cost-effectiveness evidence. Although the literature search was last updated in June 2024, overtime publication trends indicated that a further update would only yield a few additional relevant studies (~ 2–3) highly unlikely to alter any of the conclusions or improve the evidence base significantly in other ways. We also implemented gold standard systematic review methodology including a database-specific search approach for multiple electronic databases developed in collaboration with an information specialist. On the other hand, limiting studies to English and German may have excluded relevant research published in other languages. Furthermore, we faced challenges due to frequently incomplete or inconsistent reporting of relevant information across studies. Therefore, when extracting information or assigning quality scores, some potential for subjective interpretations remained. Sometimes even the specific context of palliative and/or end-of-life care was not easy to determine. By involving two researchers in the assessment process, with a third researcher resolving any disagreements, we tried to minimise the influence of subjective interpretation. Additionally, we also tried to reach out to the authors of original articles in case the reported information was unclear. Second, the broad scope made it difficult to develop a more targeted search strategy. Third, the heterogeneity of the included studies, especially concerning the different palliative and end-of-life care interventions, prevented opportunities for any quantitative synthesis or the ability to quantify the impact of specific components of care. Lastly, as for all systematic literature reviews, our findings are limited by any potential underlying publication bias, which we are not able to account for.

## Conclusions

In conclusion, there is a pressing need for high-quality economic evaluations that clearly articulate research aims and address ethical and distributional considerations. This could be supported by the development of standardised, consensus-based guidelines for conducting economic evaluations in palliative and end-of-life care settings. Such guidelines should include recommendations on broader context-relevant costs and outcomes. Additionally, current decision-making guidelines require review to allow for more flexible, context-specific value frameworks, moving beyond the standard cost-per-QALY approach. Future research should also prioritise setting and geographical diversities, as current palliative and end-of-life care assessments are exclusively from high-income and upper-middle-income countries, and concentrate mainly on hospital and home care settings. Addressing these gaps would greatly improve the evidence base, enabling future optimised palliative and end-of-life care provision.

## Supplemental Material

sj-docx-1-pmj-10.1177_02692163261418546 – Supplemental material for Economic evaluations in the palliative and end-of-life care settings: A systematic review of existing evidence, methods and quality Supplemental material, sj-docx-1-pmj-10.1177_02692163261418546 for Economic evaluations in the palliative and end-of-life care settings: A systematic review of existing evidence, methods and quality by Claudia Fischer, Elisabeth Saly, Michael Berger, Eva Katharina Masel and Judit Simon in Palliative Medicine
