# Supplementary material for: Economic evaluations in the palliative and end-of-life care settings: A systematic review of existing evidence, methods and quality
Source: Palliat Med. 2026 Feb 25;40(7):885–907. doi: 10.1177/02692163261418546 (PMC13323948; doi:10.1177/02692163261418546)
Supplement: sj-docx-1-pmj-10.1177_02692163261418546 – Supplemental material for Economic evaluations in the palliative and end-of-life care settings: A systematic review of existing evidence, methods and quality [file sj-docx-1-pmj-10.1177_02692163261418546.docx]

Appendix

**Economic evaluations in the palliative and end-of-life care settings: a systematic review of existing evidence, methods and quality**

Table of content

[List of abbreviations 2](#_Toc212587028)

[Table S1. PRISMA 2020 checklist 4](#_Toc212587029)

[Table S2. Search strategies per database 7](#_Toc212587030)

[Table S3. Overview of extracted data 9](#_Toc212587031)

[Table S4. List of studies excluded at full-text screening stage with brief reasons (n=131) 10](#_Toc212587032)

[Table S5. Additional data on the included studies (n=46) 17](#_Toc212587033)

[Figure S1. Assessment of reporting quality in included studies using the CHEC list 19](#_Toc212587034)

[References to the supplementary material 20](#_Toc212587035)

# List of abbreviations

ACP Advance Care Planning

ARG Argentina

AUS Australia

BEL Belgium

BIS Breathlessness Intervention Service

BL Baseline

BRA Brazil

BSC Best supportive care

CAN Canada

CAT COPD Assessment Test

CBA Cost-benefit analysis

CCA Cost-consequence analysis

CEA Cost-effectiveness analysis

CHE Switzerland

CHEC Consensus on Health Economic Criteria

CHEERS Consolidated Health Economic Evaluation Reporting Standards

CHF Chronic Heart Failure

CLDQ Chronic Liver Disease Questionnaire

CMA Cost-minimisation analysis

COPD Chronic obstructive pulmonary disease

CORE 34 Clinical Outcomes in Routine Evaluation

CQLQ Chinese Quality of Life Questionnaire

CSRI Client Service Receipt Inventory

CUA Cost-utility analysis

DEU Germany

DNK Denmark

EBD Endoscopic biliary drainage

EBRT External beam radiotherapy

EE Economic evaluation

EOLc End-of-life care

EORTC QLQ_C30 EORTC Core Quality of Life of Cancer Patients

EQ-5D EuroQol - 5 Dimensions – 5 Levels or 3 Levels

FACIT-Pal Functional Assessment of Chronic Illness Therapy-Palliative Care

FIMA Questionnaire for Health-Related Resource Use in an Elderly Population

FIN Finland

GAIN Gain in Alzheimer Care Instrument

GBR Great Britain

GOOSS Gastric outlet obstruction scoring system

HADS Hospital Anxiety and Depression Scale

HKG Hong Kong

ICECAP-O ICECAP Older adults

ICECAP-SCM ICECAP Supportive Care Measure

IPOS Integrated Palliative Care Outcome Scale

IST15 Isaacs Set Test (degree of cognitive impairment)

ITA Italy

iVICQ iMTA Valuation of Informal Care

JPN Japan

M Months

MFRT Multiple-fraction radiation therapy

MSCQ the MS foundation Costs Questionnaire

- Not applicable

NLD Netherlands

NRS Numerical Rating Scale

ns Not stated

NZL New Zealand

PC Palliative care

PCPD Patient Care Planning Discussions

POL Poland

POS-S-MS Palliative care Outcome Scale - Symptoms - Multiple Sclerosis

PPI Patient and Public Involvement

PTBD Percutaneous transhepaticbiliary drainage

PTC Palliative Team Care

QOD-LTC Quality of Dying - Long Term Care

QUALID Quality of Life in Late-Stage Dementia

RPP Rapid pleurodesis protocol

RT Radiation therapy

SBRT Stereotactic body radiation therapy

SF-6D Short form - 6 dimensions

SFRT Single-fraction radiation therapy

SHARED Patient Experience of Shared Decision Making

SWE Sweden

TACE Transarterial chemoembolization

THA Thailand

TIC-P Treatment Inventory of Costs in Patients with psychiatric disorders

TOPICS-MDS The Older Persons and Informal Caregivers Survey Minimum Data Set

TP Thoracoscopic talc poudrage

TPC Tunneled pleural catheter

TS Chest tube-guided talc slurry

TUR Turkey

UC Usual care

USA United States of America

# Table S1. PRISMA 2020 checklist

| **Section and Topic** | **Item #** | **Checklist item** | **Location where item is reported** |
| --- | --- | --- | --- |
| **TITLE** | | |  |
| Title | 1 | Identify the report as a systematic review. | Title page |
| **ABSTRACT** | | |  |
| Abstract | 2 | See the PRISMA 2020 for Abstracts checklist. | p. 1 |
| **INTRODUCTION** | | |  |
| Rationale | 3 | Describe the rationale for the review in the context of existing knowledge. | p. 3-5 |
| Objectives | 4 | Provide an explicit statement of the objective(s) or question(s) the review addresses. | p. 5 |
| **METHODS** | | |  |
| Eligibility criteria | 5 | Specify the inclusion and exclusion criteria for the review and how studies were grouped for the syntheses. | p. 6-7 |
| Information sources | 6 | Specify all databases, registers, websites, organisations, reference lists and other sources searched or consulted to identify studies. Specify the date when each source was last searched or consulted. | p. 6 |
| Search strategy | 7 | Present the full search strategies for all databases, registers and websites, including any filters and limits used. | Table S2 |
| Selection process | 8 | Specify the methods used to decide whether a study met the inclusion criteria of the review, including how many reviewers screened each record and each report retrieved, whether they worked independently, and if applicable, details of automation tools used in the process. | p. 7 |
| Data collection process | 9 | Specify the methods used to collect data from reports, including how many reviewers collected data from each report, whether they worked independently, any processes for obtaining or confirming data from study investigators, and if applicable, details of automation tools used in the process. | p. 7-8 |
| Data items | 10a | List and define all outcomes for which data were sought. Specify whether all results that were compatible with each outcome domain in each study were sought (e.g. for all measures, time points, analyses), and if not, the methods used to decide which results to collect. | Table S3 |
|  | 10b | List and define all other variables for which data were sought (e.g. participant and intervention characteristics, funding sources). Describe any assumptions made about any missing or unclear information. | Table S3 |
| Study risk of bias assessment | 11 | Specify the methods used to assess risk of bias in the included studies, including details of the tool(s) used, how many reviewers assessed each study and whether they worked independently, and if applicable, details of automation tools used in the process. | - |
| Effect measures | 12 | Specify for each outcome the effect measure(s) (e.g. risk ratio, mean difference) used in the synthesis or presentation of results. | - |
| Synthesis methods | 13a | Describe the processes used to decide which studies were eligible for each synthesis (e.g. tabulating the study intervention characteristics and comparing against the planned groups for each synthesis (item #5)). | - |
|  | 13b | Describe any methods required to prepare the data for presentation or synthesis, such as handling of missing summary statistics, or data conversions. | - |
|  | 13c | Describe any methods used to tabulate or visually display results of individual studies and syntheses. | p. 8 |
|  | 13d | Describe any methods used to synthesize results and provide a rationale for the choice(s). If meta-analysis was performed, describe the model(s), method(s) to identify the presence and extent of statistical heterogeneity, and software package(s) used. | p. 8 |
|  | 13e | Describe any methods used to explore possible causes of heterogeneity among study results (e.g. subgroup analysis, meta-regression). | - |
|  | 13f | Describe any sensitivity analyses conducted to assess robustness of the synthesized results. | - |
| Reporting bias assessment | 14 | Describe any methods used to assess risk of bias due to missing results in a synthesis (arising from reporting biases). | p. 8 |
| Certainty assessment | 15 | Describe any methods used to assess certainty (or confidence) in the body of evidence for an outcome. | - |
| **RESULTS** | | |  |
| Study selection | 16a | Describe the results of the search and selection process, from the number of records identified in the search to the number of studies included in the review, ideally using a flow diagram. | Figure 1 |
|  | 16b | Cite studies that might appear to meet the inclusion criteria, but which were excluded, and explain why they were excluded. | Table S4 |
| Study characteristics | 17 | Cite each included study and present its characteristics. | Table 1, Table 2, Figure 2, Table S5 |
| Risk of bias in studies | 18 | Present assessments of risk of bias for each included study. | - |
| Results of individual studies | 19 | For all outcomes, present, for each study: (a) summary statistics for each group (where appropriate) and (b) an effect estimate and its precision (e.g. confidence/credible interval), ideally using structured tables or plots. | - |
| Results of syntheses | 20a | For each synthesis, briefly summarise the characteristics and risk of bias among contributing studies. | - |
|  | 20b | Present results of all statistical syntheses conducted. If meta-analysis was done, present for each the summary estimate and its precision (e.g. confidence/credible interval) and measures of statistical heterogeneity. If comparing groups, describe the direction of the effect. | - |
|  | 20c | Present results of all investigations of possible causes of heterogeneity among study results. | - |
|  | 20d | Present results of all sensitivity analyses conducted to assess the robustness of the synthesized results. | - |
| Reporting biases | 21 | Present assessments of risk of bias due to missing results (arising from reporting biases) for each synthesis assessed. | p. 12 |
| Certainty of evidence | 22 | Present assessments of certainty (or confidence) in the body of evidence for each outcome assessed. | - |
| **DISCUSSION** | | |  |
| Discussion | 23a | Provide a general interpretation of the results in the context of other evidence. | p. 13-16 |
|  | 23b | Discuss any limitations of the evidence included in the review. | p. 16-17 |
|  | 23c | Discuss any limitations of the review processes used. | p. 16-17 |
|  | 23d | Discuss implications of the results for practice, policy, and future research. | p. 17 |
| **OTHER INFORMATION** | | |  |
| Registration and protocol | 24a | Provide registration information for the review, including register name and registration number, or state that the review was not registered. | p. 6 |
|  | 24b | Indicate where the review protocol can be accessed, or state that a protocol was not prepared. | p. 6 |
|  | 24c | Describe and explain any amendments to information provided at registration or in the protocol. | p. 6 |
| Support | 25 | Describe sources of financial or non-financial support for the review, and the role of the funders or sponsors in the review. | p. 8 |
| Competing interests | 26 | Declare any competing interests of review authors. | Title page |
| Availability of data, code and other materials | 27 | Report which of the following are publicly available and where they can be found: template data collection forms; data extracted from included studies; data used for all analyses; analytic code; any other materials used in the review. | - |

*From:*  Page MJ, McKenzie JE, Bossuyt PM, Boutron I, Hoffmann TC, Mulrow CD, et al. The PRISMA 2020 statement: an updated guideline for reporting systematic reviews. BMJ 2021;372:n71. doi: 10.1136/bmj.n71

# Table S2. Search strategies per database

| **Database: Embase, search strategy from search update in June 2024** |
| --- |
| ((('palliative nursing'/de OR 'terminally ill patient'/exp OR 'terminal care'/exp OR 'palliative therapy'/exp OR 'terminal disease'/de OR 'hospice'/de OR palliat*:ti,ab,kw OR (terminal* NEAR/6 (care* OR caring OR ill OR illness* OR patient*)):ti,ab,kw OR ('end of life':de,ti,ab,kw OR 'last year of life':de,ti,ab,kw OR 'lyol':de,ti,ab,kw OR 'life s end':de,ti,ab,kw) OR 'advanced cancer':ti,ab,kw OR hospice*:de,ti,ab,kw OR bereave*:de,ti,ab,kw) AND ('economic evaluation'/de OR 'health economics'/de OR 'economic model'/de OR 'economics'/mj OR 'health care cost'/exp/mj OR (economic* NEAR/3 (evaluat* OR aspect* OR health OR analy* OR model* OR framework* OR 'frame work*' OR method*)):ti,ab,kw OR economics:ti,kw OR (('health care' OR healthcare) NEAR/3 cost*):ti,kw)) NOT (recycl*:ti,de,kw,jt OR (waste:jt OR 'life cycle assessment':jt)) AND ([dutch]/lim OR [english]/lim OR [french]/lim OR [german]/lim OR [spanish]/lim)) NOT ((((('palliative nursing'/de OR 'terminally ill patient'/exp OR 'terminal care'/exp OR 'palliative therapy'/exp OR 'terminal disease'/de OR 'hospice'/de OR palliat*:ti,ab,kw OR (terminal* NEAR/6 (care* OR caring OR ill OR illness* OR patient*)):ti,ab,kw OR ('end of life':de,ti,ab,kw OR 'last year of life':de,ti,ab,kw OR 'lyol':de,ti,ab,kw OR 'life s end':de,ti,ab,kw) OR 'advanced cancer':ti,ab,kw OR hospice*:de,ti,ab,kw OR bereave*:de,ti,ab,kw) AND ('economic evaluation'/de OR 'health economics'/de OR 'economic model'/de OR 'economics'/mj OR 'health care cost'/exp/mj OR (economic* NEAR/3 (evaluat* OR aspect* OR health OR analy* OR model* OR framework* OR 'frame work*' OR method*)):ti,ab,kw OR economics:ti,kw OR (('health care' OR healthcare) NEAR/3 cost*):ti,kw)) NOT (recycl*:ti,de,kw,jt OR (waste:jt OR 'life cycle assessment':jt)) AND ([dutch]/lim OR [english]/lim OR [french]/lim OR [german]/lim OR [spanish]/lim)) AND ([adolescent]/lim OR [child]/lim OR [fetus]/lim OR [infant]/lim OR [newborn]/lim OR [preschool]/lim OR [school]/lim)) NOT ((((('palliative nursing'/de OR 'terminally ill patient'/exp OR 'terminal care'/exp OR 'palliative therapy'/exp OR 'terminal disease'/de OR 'hospice'/de OR palliat*:ti,ab,kw OR (terminal* NEAR/6 (care* OR caring OR ill OR illness* OR patient*)):ti,ab,kw OR ('end of life':de,ti,ab,kw OR 'last year of life':de,ti,ab,kw OR 'lyol':de,ti,ab,kw OR 'life s end':de,ti,ab,kw) OR 'advanced cancer':ti,ab,kw OR hospice*:de,ti,ab,kw OR bereave*:de,ti,ab,kw) AND ('economic evaluation'/de OR 'health economics'/de OR 'economic model'/de OR 'economics'/mj OR 'health care cost'/exp/mj OR (economic* NEAR/3 (evaluat* OR aspect* OR health OR analy* OR model* OR framework* OR 'frame work*' OR method*)):ti,ab,kw OR economics:ti,kw OR (('health care' OR healthcare) NEAR/3 cost*):ti,kw)) NOT (recycl*:ti,de,kw,jt OR (waste:jt OR 'life cycle assessment':jt)) AND ([dutch]/lim OR [english]/lim OR [french]/lim OR [german]/lim OR [spanish]/lim)) AND ([adolescent]/lim OR [child]/lim OR [fetus]/lim OR [infant]/lim OR [newborn]/lim OR [preschool]/lim OR [school]/lim)) AND ([young adult]/lim OR [adult]/lim OR [middle aged]/lim OR [aged]/lim OR [very elderly]/lim))) AND [6-6-2023]/sd |
| **Database: Health Technology Assessment - EBM Reviews (discontinued at the end of 2016)** |
| 1 Palliative Care/  2 exp Terminal Care/  3 terminally ill/  4 palliat*.mp.  5 (terminal* adj6 (care or caring or ill or illness*)).mp.  6 (end of life or last year of life or lyol or life* end).mp.  7 advanced cancer.mp.  8 hospices/  9 hospice*.mp.  10 bereave*.mp.  11 1 or 2 or 3 or 4 or 5 or 6 or 7 or 8 or 9 or 10  12 exp Health Care Costs/  13 (health care adj3 cost*).mp.  14 "Costs and Cost Analysis"/  15 Cost-Benefit Analysis/mt [Methods]  16 exp models, economic/  17 (economic* adj3 (evaluat* or aspect* or health or analy* or model* or framework* or frame work* or method*)).mp.  18 economics.mp.  19 12 or 13 or 14 or 15 or 16 or 17 or 18  20 11 and 19  21 limit 20 to "all child (0 to 18 years)"  22 limit 20 to (english or german) |
| **Database: Medline (OVID), search strategy from search update in June 2024** |
| **1**  Palliative Care/  **2**  exp Terminal Care/  **3**  Terminally Ill/  **4**  palliat*.mp.  **5**  (terminal* adj6 (care or caring or ill or illness*)).ti,ab,ot,kf.  **6**  (end of life or last year of life or lyol or life's end).ti,ab,ot,kf.  **7**  advanced cancer.ti,ab,ot,kf.  **8**  Hospices/  **9**  hospice*.ti,ab,ot,kf.  **10**  bereave*.ti,ab,ot,kf,hw.  **11**  1 or 2 or 3 or 4 or 5 or 6 or 7 or 8 or 9 or 10  **12**  exp *Health Care Costs/  **13**  ((health care or healthcare) adj3 cost*).ti,ot,kf,kw.  **14**  *"Costs and Cost Analysis"/  **15**  Cost-Benefit Analysis/mt [Methods]  **16**  exp models, economic/  **17**  (economic* adj3 (evaluat* or aspect* or health or analy* or model* or framework* or frame work* or method*)).ti,ab,ot,kf,hw.  **18**  economics.ti,ot,kf.  **19**  Palliative Care/ec  **20**  exp Terminal Care/ec  **21**  Hospices/ec  **22**  12 or 13 or 14 or 15 or 16 or 17 or 18 or 19 or 20 or 21  **23**  11 and 22  **24**  recycl*.ti,hw,kf,jw.  **25**  (waste or life cycle assessment).jw.  **26**  24 or 25  **27**  23 not 26  **28**  limit 27 to (dutch or english or german or french or spanish)  **29**  limit 28 to "all child (0 to 18 years)"  **30**  limit 29 to "all adult (19 plus years)"  **31**  29 not 30  **32**  28 not 31  **33**  limit 32 to dt=20230606-20240604  **34**  limit 32 to ed=20230606-20240604  **35**  33 or 34 |
| **Database: NHS Economic Evaluation Database - EBM Reviews (discontinued at the beginning of 2016)** |
| 1 Palliative Care/  2 exp Terminal Care/  3 terminally ill/  4 palliat*.mp. [mp=title, text, subject heading word]  5 (terminal* adj6 (care or caring or ill or illness*)).mp. [mp=title, text, subject heading word]  6 (end of life or last year of life or lyol or life* end).mp. [mp=title, text, subject heading word]  7 advanced cancer.mp. [mp=title, text, subject heading word]  8 hospices/  9 hospice*.mp. [mp=title, text, subject heading word]  10 bereave*.mp. [mp=title, text, subject heading word]  11 1 or 2 or 3 or 4 or 5 or 6 or 7 or 8 or 9 or 10  12 limit 11 to "all child (0 to 18 years)"  13 limit 12 to "all adult (19 plus years)"  14 12 not 13  15 11 not 14  16 limit 15 to yr="2010 - 2015" |

# Table S3. Overview of extracted data

| ***Category*** | **Data and description** |
| --- | --- |
| *Descriptive study characteristics* | First author, year/journal of publication, country/countries of data origin, study type |
| *Population characteristics* | Total sample size of the study population, disease area, and the perceived type of care (end-of-life and/or palliative) |
| *Key methodological features* | Intervention(s) and comparator, perceived type of economic evaluation, settings (e.g., hospital or hospice), applied time horizon (the duration over which health outcomes and costs were calculated), and discount rates for outcomes and costs separately |
| *Outcome methods* | Population of interest (for whom the outcomes were measured), the specific utility measurement instruments were noted for cost-utility analyses, along with whether the instrument was generic, palliative care-specific, or disease-specific (e.g., cancer-specific). For other analyses, we reported the outcome measures utilized. The methods of data collection were categorized based on the assessment with instruments: We differentiated between self-reported, assisted, and proxy methods. |
| *Costing methods* | The costing perspective was classified according to the type of costs considered in the economic evaluation (e.g., healthcare or family costs) We extracted the sources of resource use, which could include for instance administrative data or patient-reported data. The sources of unit costs were categorized into unit cost databases, administrative data, published studies and reports, tariffs, expert opinion, study-specific calculations, or unspecified sources. |
| *Cost-effectiveness results* | We categorized the results as dominant, cost-effective (minimum 50% probability, if reported), not cost-effective (less than 50% probability, if reported), dominated, uncertain due to results, and unclear due to incomplete reporting (e.g., missing threshold information) as per reported information. |

# Table S4. List of studies excluded at full-text screening stage with brief reasons (n=131)

| **Author** | **Year** | **Title** | **Exclusion reason** |
| --- | --- | --- | --- |
| Adejumo et al. | 2020 | Suboptimal Use of Inpatient Palliative Care Consultation May Lead to Higher Readmissions and Costs in End-Stage Liver Disease | Other study type (e.g., no full economic evaluation) |
| Akhtar et al. | 2020 | Characterizing the Financial Value of In-Home Palliative Care for Patients, Payers, and Hospitals | Other study type (e.g., no full economic evaluation) |
| Alemao et al. | 2015 | Cost effectiveness analysis of abatacept compared with adalimumab on background methotrexate in biologic-naive ra adult patients by anti-cyclic citrullinated peptide-positive subgroups | Other publication type (e.g., conference abstract, comment) |
| Aoun et al. | 2023 | The compassionate communities connectors program: effect on healthcare usage | Other study type (e.g., no full economic evaluation) |
| Ayyagari et al. | 2016 | Economic evaluation of octreotide LAR versus lanreotide depot in the treatment of metastatic gastrointestinal neuroendocrine tumors | Other study type (e.g., no full economic evaluation) |
| Bernstein et al. | 2018 | Higher Quality, Lower Cost with an Innovative Geriatrics Consultation Service | Other setting (not palliative or end-of-life care) |
| Bewersdorf et al. | 2022 | Cost-Effectiveness of Azacitidine and Ivosidenib in Newly Diagnosed Older, Intensive Chemotherapy-Ineligible Patients with IDH1-Mutant Acute Myeloid Leukemia | Other setting (not palliative or end-of-life care) |
| Bonastre et al. | 2012 | Metastatic breast cancer: we do need primary cost data | Other study type (e.g., no full economic evaluation) |
| Bradford et al. | 2014 | Paediatric palliative care by video consultation at home: a cost minimisation analysis | Other patient population (e.g., children, adolescents) |
| Brick et al. | 2017 | Costs of formal and informal care in the last year of life for patients in receipt of specialist palliative care | Other study type (e.g., no full economic evaluation) |
| Bryant | 2016 | Original Canadian cancer costing research for cancer control sustainability, quality, and value | Other study type (e.g., no full economic evaluation) |
| Buchanan et al. | 2017 | Using Genomic Information to Guide Ibrutinib Treatment Decisions in Chronic Lymphocytic Leukaemia: A Cost-Effectiveness Analysis | Other patient population (e.g., children, adolescents) |
| Budzynska et al. | 2016 | Comparison of patency and cost-effectiveness of self-expandable metal and plastic stents used for malignant biliary strictures: a Polish single-center study | Other setting (not palliative or end-of-life care) |
| Bullement et al. | 2018 | Cost-effectiveness of Trifluridine/tipiracil for Previously Treated Metastatic Colorectal Cancer in England and Wales | Other setting (not palliative or end-of-life care) |
| Burki | 2016 | Counting the cost of end-of-life cancer care | Other publication type (e.g., conference abstract, comment) |
| Cartoni et al. | 2015 | Hospital versus home care for patients with hematological malignancies in curative or terminal phase: Use of the resources analysis, symptom burden and cost-effectiveness study | Other publication type (e.g., conference abstract, comment) |
| Casciano et al. | 2010 | Economic evaluation of everolimus versus sorafenib for the treatment of advanced renal cell carcinoma after failure on treatment with sunitinib | Other setting (not palliative or end-of-life care) |
| Chaudhuri et al. | 2017 | Critical care at the end of life: a population-level cohort study of cost and outcomes | Other study type (e.g., no full economic evaluation) |
| Chiang et al. | 2016 | Impact of Home Hospice Care on Patients with Advanced Lung Cancer: A Longitudinal Population-Based Study in Taiwan | Other study type (e.g., no full economic evaluation) |
| Chiang et al. | 2019 | Comparison of medical outcomes and health care costs at the end of life between dialysis patients with and without cancer: a national population-based study | Other study type (e.g., no full economic evaluation) |
| Crespo et al. | 2013 | Cost-effectiveness analysis of azacitidine in the treatment of high-risk myelodysplastic syndromes in Spain | Other setting (not palliative or end-of-life care) |
| Cummins et al. | 2012 | Cost effectiveness of golimumab for the treatment of active psoriatic arthritis | Other patient population (e.g., children, adolescents) |
| Cummins et al. | 2011 | Cost-effectiveness of infliximab for the treatment of active and progressive psoriatic arthritis | Other setting (not palliative or end-of-life care) |
| Curtis et al. | 2016 | Randomized Trial of Communication Facilitators to Reduce Family Distress and Intensity of End-of-Life Care | Other study type (e.g., no full economic evaluation) |
| Dinan et al. | 2016 | Resource Use in the Last Year of Life Among Patients Who Died With Versus of Prostate Cancer | Other study type (e.g., no full economic evaluation) |
| Fitzpatrick et al. | 2018 | Economic impact of early inpatient palliative care intervention in a community hospital setting | Other study type (e.g., no full economic evaluation) |
| Gabrio | 2021 | A Bayesian Framework for Patient-Level Partitioned Survival Cost-Utility Analysis | Other study type (e.g., no full economic evaluation) |
| Glickman et al. | 2024 | Costing a Nurse and Social Worker Palliative Telecare Intervention for People with Chronic Heart and Lung Diseases (RP304) | Other publication type (e.g., conference abstract, comment) |
| González et al. | 2017 | Efficiency of nivolumab in the treatment of second-line advanced non-squamous non-small cell lung cancer (NSCLC) in Spain | Other publication type (e.g., conference abstract, comment) |
| Goodall et al. | 2014 | Cost-effectiveness of colonic stents for the management of malignant large bowel obstruction | Other publication type (e.g., conference abstract, comment) |
| Gordon et al. | 2021 | Home Palliative Care Savings | Other study type (e.g., no full economic evaluation) |
| Graham et al. | 2016 | Economic Analysis of Panitumumab Compared With Cetuximab in Patients With Wild-type KRAS Metastatic Colorectal Cancer That Progressed After Standard Chemotherapy | Other setting (not palliative or end-of-life care) |
| Greenhalgh et al. | 2010 | Pemetrexed for the maintenance treatment of locally advanced or metastatic non-small cell lung cancer | Other setting (not palliative or end-of-life care) |
| Guadagnolo et al. | 2013 | Use of radiation therapy in the last 30 days of life among a large population-based cohort of elderly patients in the United States | Other study type (e.g., no full economic evaluation) |
| Guadagnolo et al. | 2014 | Increasing use of advanced radiation therapy technologies in the last 30 days of life among patients dying as a result of cancer in the United States | Other study type (e.g., no full economic evaluation) |
| Guerriere et al. | 2010 | Cost variations in ambulatory and home-based palliative care | Other study type (e.g., no full economic evaluation) |
| Hartwell et al. | 2011 | Topotecan for relapsed small cell lung cancer: a systematic review and economic evaluation | Other setting (not palliative or end-of-life care) |
| Heathfield et al. | 2016 | Quantitate, qualitative and economic review of marie curie integrated palliative care service | Other publication type (e.g., conference abstract, comment) |
| Hess et al. | 2012 | Cost of palliative radiation to the bone for patients with bone metastases secondary to breast or prostate cancer | Other study type (e.g., no full economic evaluation) |
| Higginson et al. | 2020 | Associations between informal care costs, care quality, carer rewards, burden and subsequent grief: the international, access, rights and empowerment mortality follow-back study of the last 3 months of life (IARE I study) | Other study type (e.g., no full economic evaluation) |
| Hopkins et al. | 2015 | Cost effectiveness of ex vivo lung perfusion warrants analysis of long term recipient outcome and donor organ utilization rate | Other publication type (e.g., conference abstract, comment) |
| Huang et al. | 2020 | Differences in medical costs for end-of-life patients receiving traditional care and those receiving hospice care: A retrospective study | Other study type (e.g., no full economic evaluation) |
| Hudson et al. | 2021 | Do family meetings for hospitalised palliative care patients improve outcomes and reduce health care costs? A cluster randomised trial | Other study type (e.g., no full economic evaluation) |
| Huo et al. | 2020 | Timing, costs, and survival outcome of specialty palliative care in medicare beneficiaries with metastatic non-small-cell lung cancer | Other study type (e.g., no full economic evaluation) |
| Hwang et al. | 2013 | Hospice offers more palliative care but costs less than usual care for terminal geriatric hepatocellular carcinoma patients: a nationwide study | Other study type (e.g., no full economic evaluation) |
| Isenberg et al. | 2017 | Economic Evaluation of a Hospital-Based Palliative Care Program | Other study type (e.g., no full economic evaluation) |
| Isenberg et al. | 2020 | Cost-effectiveness of Investment in End-of-Life Home Care to Enable Death in Community Settings | Other study type (e.g., no full economic evaluation) |
| Isenberg et al. | 2019 | Evaluating the Impact and Costs of Home-Based Palliative Care at the System Level (FR420C) | Other publication type (e.g., conference abstract, comment) |
| Isshiki | 2014 | HPV vaccination for cervical cancer prevention is not cost-effective in Japan | Other setting (not palliative or end-of-life care) |
| Javanbakht et al. | 2022 | Cost-effectiveness analysis of pressurized intraperitoneal aerosol chemotherapy (PIPAC) in patients with gastric cancer and peritoneal metastasis | Other setting (not palliative or end-of-life care) |
| Jayatunga et al. | 2019 | Health and social care costs at the end of life: a matched analysis of linked patient records in East London | Other study type (e.g., no full economic evaluation) |
| Jones et al. | 2015 | Cost effectiveness analysis of eribulin mesylate (Halaven®) as a treatment for metastatic breast cancer in Mexico | Other publication type (e.g., conference abstract, comment) |
| Kapol et al. | 2016 | Economic evaluation of pegylated interferon plus ribavirin for treatment of chronic hepatitis C in Thailand: genotype 1 and 6 | Other patient population (e.g., children, adolescents) |
| Kim et al. | 2016 | Cost-effectiveness of palliative surgery versus nonsurgical procedures in gastrointestinal cancer patients | Other study type (e.g., no full economic evaluation) |
| Kolbin et al. | 2010 | Pharmacoepidemiological and pharmacoeconomic evaluation of oxaliplatin in palliative chemotherapy of metastatic colorectal cancer (mCCR) | Other publication type (e.g., conference abstract, comment) |
| Kondo et al. | 2022 | Cost-effectiveness analysis of pegfilgrastim in patients with non-small cell lung cancer receiving ramucirumab plus docetaxel in Japan | Other setting (not palliative or end-of-life care) |
| Konidaris et al. | 2021 | Assessing the Value of Cemiplimab for Adults With Advanced Cutaneous Squamous Cell Carcinoma: A Cost-Effectiveness Analysis | Other setting (not palliative or end-of-life care) |
| Konski et al. | 2011 | Feasibility of economic analysis of Radiation Therapy Oncology Group (RTOG) 91-11 using Medicare data | Other setting (not palliative or end-of-life care) |
| Kourlaba et al. | 2015 | Economic evaluation of trimetazidine in the management of chronic stable angina in Greece | Other setting (not palliative or end-of-life care) |
| Kozhevnikov et al. | 2019 | Implementing an emergency department EMR trigger tool for palliative medicine consultation and its effect on length of stay and health care costs: A retrospective study | Other publication type (e.g., conference abstract, comment) |
| Lee et al. | 2014 | A cost-utility analysis of degarelix in the treatment of advanced hormone-dependent prostate cancer in the United Kingdom | Other patient population (e.g., children, adolescents) |
| Lee et al. | 2011 | Cost effectiveness of bevacizumab plus folfiri versus folfiri in treatment of advanced metastatic colorectal cancer in the republic of Korea | Other publication type (e.g., conference abstract, comment) |
| Lehn et al. | 2019 | Pharmacists Providing Palliative Care Services: Demonstrating a Positive Return on Investment | Other study type (e.g., no full economic evaluation) |
| Lomenick et al. | 2021 | Economics of Using Telemedicine to Supplement Hospice Care in Rural Areas | Other study type (e.g., no full economic evaluation) |
| Lux et al. | 2010 | Results of the Zometa cost-utility model for the german healthcare system based on the results of the ABCSG-12 study | Other setting (not palliative or end-of-life care) |
| Maessen et al. | 2024 | An economic evaluation of an early palliative care intervention among patients with advanced cancer | Other study type (e.g., no full economic evaluation) |
| Maessen et al. | 2021 | Is early palliative care associated with reduced health care cost of end-of-life care in patients with advanced cancer? | Other publication type (e.g., conference abstract, comment) |
| Majethia et al. | 2015 | Cost effectiveness analysis of eribulin mesylate as a treatment for metastatic breast cancer in spain: Management in the later lines of therapy | Other publication type (e.g., conference abstract, comment) |
| Malayev et al. | 2012 | Palliative chemotherapy for malignant ascites secondary to ovarian cancer | Other study type (e.g., no full economic evaluation) |
| Manfredi et al. | 2021 | Early palliative intervention in septic patients reduces healthcare utilization | Other study type (e.g., no full economic evaluation) |
| Maran et al. | 2012 | Cost effectiveness analysis in the veneto region of nab-paclitaxel monotherapy ii line versus conventional paclitaxel in patients with breast cancer and for whom anthracyclines are not indicated | Other publication type (e.g., conference abstract, comment) |
| Marcellusi et al. | 2012 | Economic evaluation of adalimumab versus other biologic treatments for moderate to severe psoriatic arthritis in Italy | Other publication type (e.g., conference abstract, comment) |
| Marchetti et al. | 2018 | Cost-effectiveness of axicabtagene ciloleucel for relapsed or refractory dffuse large b-cell lymphoma in Italy | Other publication type (e.g., conference abstract, comment) |
| Mc Caffrey et al. | 2010 | The old dog & the poor relation: Informing funding decisions in palliative care with cost-consequence analysis in the cost-disutility plane | Other publication type (e.g., conference abstract, comment) |
| McCarthy et al. | 2024 | Cost-effectiveness of pembrolizumab for previously treated MSI-H/dMMR solid tumours in the UK | Other setting (not palliative or end-of-life care) |
| McQueen et al. | 2022 | Cost and Return on Investment of a Team-Based Palliative Care Program for Parkinson Disease | Other study type (e.g., no full economic evaluation) |
| Mervin | 2016 | Cost-effectiveness of transfemoral aortic valve implantation compared with surgical aortic valve and medical management in patients with severe symptomatic aortic stenosis in Australia | Other publication type (e.g., conference abstract, comment) |
| Miller et al. | 2017 | POEM is a cost-effective procedure: cost-utility analysis of endoscopic and surgical treatment options in the management of achalasia | Other setting (not palliative or end-of-life care) |
| Miranda et al. | 2021 | Improved quality of care and reduced healthcare costs at the end-of-life among older people with dementia who received palliative home care: A nationwide propensity score-matched decedent cohort study | Other study type (e.g., no full economic evaluation) |
| Mitchell et al. | 2020 | Exploring the costs, consequences and efficiency of three types of palliative care day services in the UK: a pragmatic before-and-after descriptive cohort study | Other study type (e.g., no full economic evaluation) |
| Monnery et al. | 2023 | Delivery Models and Health Economics of Supportive Care Services in England: A Multicentre Analysis | Other study type (e.g., no full economic evaluation) |
| Moreton et al. | 2020 | Economic and clinical outcomes of the nurse practitioner-led Sydney Adventist Hospital Community Palliative Care Service | Other study type (e.g., no full economic evaluation) |
| Morrison | 2015 | Economic evaluation of specialist inpatient palliative care consultation teams: Cost effect estimates vary by treatment timeliness | Other publication type (e.g., conference abstract, comment) |
| Morrison | 2015 | Economic evaluation of specialist inpatient palliative care consultation teams: Treatment effect varies by patient complexity | Other publication type (e.g., conference abstract, comment) |
| Nam et al. | 2018 | Economic evaluation of rituximab in addition to standard of care chemotherapy for adult patients with acute lymphoblastic leukemia | Other setting (not palliative or end-of-life care) |
| Nguyen et al. | 2017 | An economic model of advance care planning in Australia: a cost-effective way to respect patient choice | Other setting (not palliative or end-of-life care) |
| Norman et al. | 2010 | Capecitabine for the treatment of advanced gastric cancer | Other setting (not palliative or end-of-life care) |
| Pace et al. | 2012 | Quality of care and rehospitalization rate in the last stage of disease in brain tumor patients assisted at home: a cost effectiveness study | Other study type (e.g., no full economic evaluation) |
| Park et al. | 2019 | Cost-Utility Analysis of Sacubitril/Valsartan Use Compared With Standard Care in Chronic Heart Failure Patients With Reduced Ejection Fraction in South Korea | Other setting (not palliative or end-of-life care) |
| Parker et al. | 2023 | Cost-Effectiveness of Lisocabtagene Maraleucel Versus Axicabtagene Ciloleucel and Tisagenlecleucel in the Third-Line or Later Treatment Setting for Relapsed or Refractory Large B-cell Lymphoma in the United States | Other setting (not palliative or end-of-life care) |
| Patel et al. | 2020 | Cost-effectiveness of first-line vs third-line ibrutinib in patients with untreated chronic lymphocytic leukemia | Other setting (not palliative or end-of-life care) |
| Pereira et al. | 2020 | Integrated palliative homecare in advanced dementia: reduced healthcare utilisation and costs | Other study type (e.g., no full economic evaluation) |
| Perrin et al. | 2015 | Lifetime cost of everolimus vs axitinib in patients with advanced renal cell carcinoma who failed prior sunitinib therapy in the US | Other study type (e.g., no full economic evaluation) |
| Porte et al. | 2024 | Cost-effectiveness of avelumab first-line maintenance therapy for adult patients with locally advanced or metastatic urothelial carcinoma in France | Other setting (not palliative or end-of-life care) |
| Powers et al. | 2015 | Cost savings associated with expanded hospice use in Medicare | Other study type (e.g., no full economic evaluation) |
| Ramaekers et al. | 2018 | Trifluridine-Tipiracil for Previously Treated Metastatic Colorectal Cancer: An Evidence Review Group Perspective of a NICE Single Technology Appraisal | Other study type (e.g., no full economic evaluation) |
| Rao et al. | 2024 | Effects of Primary Care-Led, Integrated Palliative Care for Medicare Patients in a Value-Based Model | Other study type (e.g., no full economic evaluation) |
| Ross et al. | 2018 | Spiritual support in end stage heart failure (ESHF): A randomised controlled feasibility study | Other publication type (e.g., conference abstract, comment) |
| Round et al. | 2014 | A cost-utility analysis of a rehabilitation service for people living with and beyond cancer | Other setting (not palliative or end-of-life care) |
| Schueler et al. | 2020 | Cost-Effectiveness of a Small Intrapericardial Centrifugal LVAD versus Medical Management in Destination Therapy Patients in the UK | Other publication type (e.g., conference abstract, comment) |
| Serreli et al. | 2012 | The effects of palliative care consultations on hospital costs and family satisfaction | Other publication type (e.g., conference abstract, comment) |
| Singh et al. | 2013 | Evaluation of the role and cost-effectiveness of end-of-life orthopaedic interventions in cancer patients with skeletal metastases to the hip | Other study type (e.g., no full economic evaluation) |
| Skedgel et al. | 2011 | Is adjuvant trastuzumab economically justified in Her-2/neu positive T1bNO breast cancer? | Other publication type (e.g., conference abstract, comment) |
| Sofrygin et al. | 2010 | Cost-effectiveness of lapatinib plus capecitabine (LAP+C) versus capecitabine alone (C-only) or trastuzumab plus capecitabine (TZ+C) in women with HER2-positive metastatic breast cancer (MBC) who have received prior therapy with trastuzumab (TZ) from the U.K. National Health Service (NHS) perspective | Other setting (not palliative or end-of-life care) |
| Soto Molina et al. | 2014 | Cost-effectiveness analysis of oxycodone lp an opioid analgesic for patients with moderate to severe pain secondary to cancer in Mexico | Other publication type (e.g., conference abstract, comment) |
| Spackman et al. | 2013 | Trastuzumab for the treatment of HER2-positive metastatic gastric cancer : a NICE single technology appraisal | Other study type (e.g., no full economic evaluation) |
| Stewart et al. | 2022 | Cancer centre supportive oncology service: health economic evaluation | Other study type (e.g., no full economic evaluation) |
| Su et al. | 2023 | A cost-effectiveness analysis of avelumab plus best supportive care versus best supportive care alone as first-line maintenance treatment for patients with locally advanced or metastatic urothelial carcinoma in Taiwan | Other setting (not palliative or end-of-life care) |
| Sudat et al. | 2018 | Impact of home-based, patient-centered support for people with advanced illness in an open health system: A retrospective claims analysis of health expenditures, utilization, and quality of care at end of life | Other study type (e.g., no full economic evaluation) |
| Sullivan et al. | 2016 | The cost effectiveness of idelalisib in chronic lymphocytic leukaemia in England and Wales | Other publication type (e.g., conference abstract, comment) |
| Swallow et al. | 2018 | The Additional Costs per Month of Progression-Free Survival and Overall Survival: An Economic Model Comparing Everolimus with Cabozantinib, Nivolumab, and Axitinib for Second-Line Treatment of Metastatic Renal Cell Carcinoma | Other setting (not palliative or end-of-life care) |
| Tanguy-Melac et al. | 2020 | Intensity of Care, Expenditure, and Place of Death in French Women in the Year Before Their Death From Breast Cancer: A Population-Based Study | Other publication type (e.g., conference abstract, comment) |
| Tholomier et al. | 2020 | Cost-effectiveness of immunotherapies in metastatic bladder cancer: A Canadian healthcare perspective | Other publication type (e.g., conference abstract, comment) |
| Thomas et al. | 2013 | Outcome of patients treated with a single-fraction dose of palliative radiation for cutaneous T-cell lymphoma | Other study type (e.g., no full economic evaluation) |
| Tikhonova et al. | 2017 | Azacitidine for Treating Acute Myeloid Leukaemia with More Than 30 % Bone Marrow Blasts: An Evidence Review Group Perspective of a National Institute for Health and Care Excellence Single Technology Appraisal | Other study type (e.g., no full economic evaluation) |
| Tikhonova et al. | 2018 | Economic Analysis of First-Line Treatment with Cetuximab or Panitumumab for RAS Wild-Type Metastatic Colorectal Cancer in England | Other setting (not palliative or end-of-life care) |
| Tikhonova et al. | 2016 | Cost effectiveness of cetuximab and panitumumab for first-line ras WT metastatic colorecal cancer | Other publication type (e.g., conference abstract, comment) |
| Tremblay et al. | 2019 | PCN49 COST-EFFECTIVENESS MODEL COMPARING EXPANDED UMBILICAL CORD BLOOD TRANSPLANTS WITH OTHER HSCT STRATEGIES FOR PATIENTS WITH HAEMATOLOGICAL MALIG-NCIES | Other publication type (e.g., conference abstract, comment) |
| Tremblay et al. | 2016 | Cost-effectiveness analysis of lenvatinib as a treatment for radioactive iodine refractory differentiated thyroid cancer in The United States | Other publication type (e.g., conference abstract, comment) |
| Tsukiyama et al. | 2017 | A Cost-Effectiveness Analysis of Gemcitabine plus Cisplatin Versus Gemcitabine Alone for Treatment of Advanced Biliary Tract Cancer in Japan | Other setting (not palliative or end-of-life care) |
| Urwin et al. | 2021 | The monetary valuation of informal care to cancer decedents at end-of-life: Evidence from a national census survey | Other study type (e.g., no full economic evaluation) |
| Wang et al. | 2017 | An economic evaluation model for follicular lymphoma (FL): Predicting treatment cost, life expectancy and quality-adjusted life year of different scenarios using UK population based observational data | Other publication type (e.g., conference abstract, comment) |
| Weckmann MT et al. | 2013 | Medical manuscripts impact of hospice enrollment on cost and length of stay of a terminal admission | Other study type (e.g., no full economic evaluation) |
| Weinstein et al. | 2022 | Quality and cost outcomes of an integrated supportive care program | Other study type (e.g., no full economic evaluation) |
| Wolf et al. | 2019 | Clinical pharmacists in palliative care: effects on drug therapy and drug expenses | Other study type (e.g., no full economic evaluation) |
| Wu et al. | 2011 | Economic evaluation of sunitinib malate for the first-line treatment of metastaric renal cell carcinoma in the chinese health care setting | Other publication type (e.g., conference abstract, comment) |
| Xie et al. | 2011 | Economic evaluation of denosumab compared with zoledronic acid in hormone-refractory prostate cancer patients with bone metastases | Other setting (not palliative or end-of-life care) |
| Yang et al. | 2012 | Golimumab for the treatment of psoriatic arthritis: a NICE single technology appraisal | Other study type (e.g., no full economic evaluation) |
| Yosick et al. | 2019 | Effects of a Population Health Community-Based Palliative Care Program on Cost and Utilization | Other study type (e.g., no full economic evaluation) |
| Yu et al. | 2015 | Societal costs of home and hospital end-of-life care for palliative care patients in Ontario, Canada | Other study type (e.g., no full economic evaluation) |
| Zhou et al. | 2022 | Economic Evaluation of Sintilimab Plus Bevacizumab Versus Sorafenib as a First-line Treatment for Unresectable Hepatocellular Carcinoma | Other setting (not palliative or end-of-life care) |

# Table S5. Additional data on the included studies (n=46)

| **Author** | **Year** | **Discounting** | | **Utility measurement tool** | **Data collection interval** | **Data collection frequency** | **Tool for resource use data collection** |
| --- | --- | --- | --- | --- | --- | --- | --- |
|  |  | **Outcomes** | **Costs** |  |  |  |  |
| Bauer et al.^63^ | 2020 | - | - | Generic | - | - | - |
| Bennett et al.^64^ | 2017 | - | - | Generic | Biweekly | 4x | - |
| Cartoni et al.^65^ | 2021 | ns | ns | - | BL, 1, 2, 4 weeks | 4x | - |
| Chang et al.^66^ | 2020 | 3% | 3% | - | - | - | - |
| Collinson et al.^67^ | 2016 | 3% | 3% | Generic | - | - | - |
| Earnshaw et al.^68^ | 2010 | - | - | Generic | - | - | - |
| El Alili et al.^69^ | 2020 | ns | - | Generic | BL, 1, 3, 6, 12 months | 5x | TOPICS-MDS (adapted) |
| El Alili et al.^70^ | 2020 | ns | - | Generic | BL, 3, 10, 24, 48 weeks | 5x | TiC-P (adapted) |
| Evans et al.^71^ | 2021 | ns | ns | Generic | BL, 6, 12 weeks | 3x | CSRI |
| Farquhar et al.^72^ | 2014 | ns | ns | Generic | 1, 3, 5 weeks | 3x | CSRI |
| Farquhar et al.^73^ | 2016 | ns | ns | Generic | BL, 2, 4 weeks | 3x | CSRI |
| Froggatt et al.^74^ | 2020 | ns | - | - | Biweekly | 3x | CSRI (adapted) |
| Furlan et al.^75^ | 2012 | ns | ns | ns | - | - | - |
| Gottschalk et al.^76^ | 2023 | - | - | Generic | BL, 6, 12, 24, 48 weeks | 5x | FIMA (adapted) |
| Halling et al.^77^ | 2020 | ns | ns | Generic, disease-specific | BL, 2, 4, 8 weeks, 6 months | 5x | iVICQ |
| Hashimoto et al.^78^ | 2021 | ns | ns | - | Once a day or more | Different per patient | - |
| Huo et al.^79^ | 2014 | - | - | - | - | - | - |
| Iskedjian et al.^80^ | 2011 | ns | ns | - | - | - | - |
| Jeurnink et al.^81^ | 2010 | ns | ns | - | BL, 2, 4 weeks, monthly until death | Different per patient | - |
| Johnson et al.^82^ | 2015 | ns | ns | Generic | BL, 1, 2, 3, 4, 8 weeks | 6x | - |
| Jones et al.^83^ | 2013 | - | - | Generic | BL, 3 months | 2x | CSRI (adapted) |
| Kim et al.^84^ | 2014 | 3% | 3% | ns | - | - | - |
| Lamfre et al.^85^ | 2024 | - | - | - | - | - | - |
| Ljungman et al.^86^ | 2013 | 5% | 5% | - | - | - | - |
| Lowery et al.^87^ | 2013 | ns | ns | ns | - | - | - |
| McCaffrey et al.^88^ | 2013 | - | - | - | ns | ns | - |
| McCaffrey et al.^89^ | 2019 | - | - | - | ns | ns | - |
| Meads et al.^90^ | 2019 | - | - | Generic | - | - | Questionnaire |
| O’Halloran et al.^91^ | 2020 | - | - | - | BL, 12 weeks | 2x | Cost diary |
| Pattenden et al.^92^ | 2012 | ns | ns | - | ns | ns | - |
| Pham et al.^93^ | 2014 | - | - | Generic | - | - | - |
| Rosato et al.^94^ | 2021 | ns | ns | Generic | BL, 3, 6 months | 3x | MSCQ |
| Sahakyan et al.^95^ | 2023 | - | - | Generic | BL, 1, 2, 3, 4, 5, 6, 9, 12 months | 9x | Cost diary |
| Sahlen et al.^96^ | 2016 | ns | ns | Generic | BL, 6 months | 2x | - |
| Sangmala et al.^97^ | 2018 | 3% | 3% | Generic | - | - | - |
| Saygili et al.^98^ | 2019 | ns | ns | - | ns | ns | - |
| Sellars et al.^99^ | 2022 | ns | ns | - | - | - | - |
| Shafiq et al.^100^ | 2015 | - | - | Generic | - | - | - |
| Suttichaimongkol et al.^101^ | 2018 | ns | ns | Generic | - | 1x | - |
| Thein et al.^102^ | 2017 | 3% | 3% | ns | - | - | - |
| Verberkt et al.^103^ | 2021 | - | - | Generic | BL, 4 weeks | 2x | Cost diary |
| Vieira et al.^104^ | 2024 | - | - | - | BL, 60 days, end of phase 2 and phase 3 | 4x | Interview |
| Wichmann et al.^105^ | 2020 | - | ns | - | BL, 13, 17 months | 3x | - |
| Wong et al.^106^ | 2018 | ns | ns | Generic | BL, 28, 84 days | 3x | - |
| Wu et al.^107^ | 2021 | ns | ns | Generic | ns | ns | - |
| Yi et al.^108^ | 2022 | 3.5% | 3.5% | Generic | - | - | - |

Abbreviations (in alphabetical order): BL = Baseline, CSRI = Client Service Receipt Inventory, FIMA = Questionnaire for Health-Related Resource Use in an Elderly Population, iVICQ = iMTA Valuation of Informal Care, MSCQ = the MS foundation Costs Questionnaire, ns = not stated, TIC-P = Treatment Inventory of Costs in Patients with psychiatric disorders, TOPICS-MDS = The Older Persons and Informal Caregivers Survey Minimum Data Set, x = time(s), ‘-‘ = Not applicable

# Figure S1. Assessment of reporting quality in included studies using the CHEC list


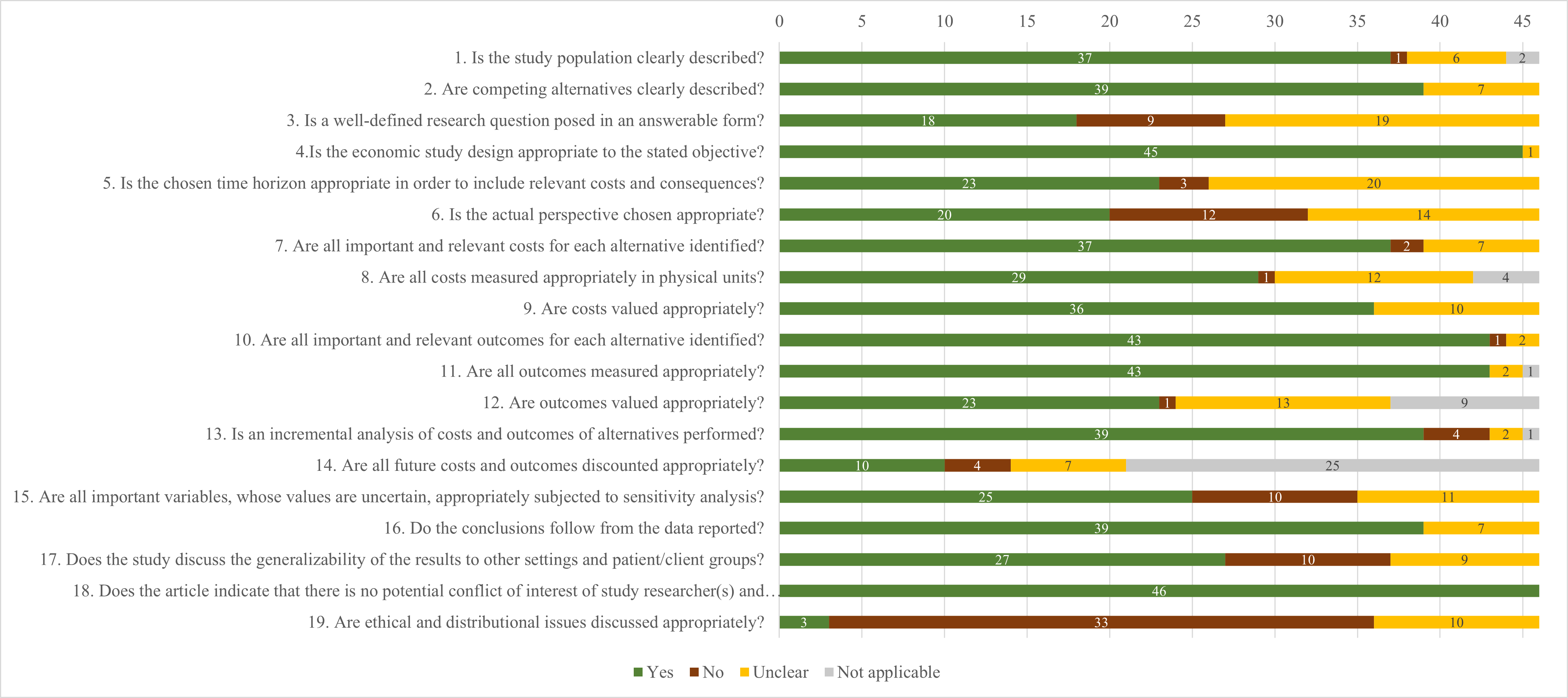


# References to the supplementary material

63. Bauer A, Dixon J, Knapp M, et al. Exploring the cost-effectiveness of advance care planning (by taking a family carer perspective): Findings of an economic modelling study. Health Soc Care Community 2021; 29: 967–981. DOI: 10.1111/hsc.13131.

64. Bennett MI, Mulvey MR, Campling N, et al. Self-management toolkit and delivery strategy for end-of-life pain: the mixed-methods feasibility study. Health Technology Assessment (Winchester, England) 2017.

65. Cartoni C, Breccia M, Giesinger JM, et al. Early Palliative Home Care versus Hospital Care for Patients with Hematologic Malignancies: A Cost-Effectiveness Study. J Palliat Med 2021; 24: 887–893. DOI: 10.1089/jpm.2020.0396.

66. Chang EM, Shaverdian N, Capiro N, et al. Cost Effectiveness of External Beam Radiation Therapy versus Percutaneous Image-Guided Cryoablation for Palliation of Uncomplicated Bone Metastases. J Vasc Interv Radiol 2020; 31: 1221–1232. DOI: 10.1016/j.jvir.2020.03.027.

67. Collinson L, Kvizhinadze G, Nair N, et al. Economic evaluation of single-fraction versus multiple-fraction palliative radiotherapy for painful bone metastases in breast, lung and prostate cancer. J Med Imaging Radiat Oncol 2016; 60: 650–660. DOI: 10.1111/1754-9485.12467.

68. Earnshaw SR, Klok RM, Iyer S, et al. Methylnaltrexone bromide for the treatment of opioid-induced constipation in patients with advanced illness – a cost-effectiveness analysis. Aliment Pharmacol Ther 2010; 31: 911–921. DOI: 10.1111/j.1365-2036.2010.04244.x.

69. El Alili M, Smaling HJ, Joling KJ, et al. Cost-effectiveness of the Namaste care family program for nursing home residents with advanced dementia in comparison with usual care: a cluster-randomized controlled trial. BMC Health Serv Res 2020; 20: 831. DOI: 10.1186/s12913-020-05570-2.

70. El Alili M, Schuurhuizen Csew, Braamse AMJ, et al. Economic evaluation of a combined screening and stepped-care treatment program targeting psychological distress in patients with metastatic colorectal cancer: A cluster randomized controlled trial. Palliat Med 2020; 34: 934–945. DOI: 10.1177/0269216320913463.

71. Evans CJ, Bone AE, Yi D, et al. Community-based short-term integrated palliative and supportive care reduces symptom distress for older people with chronic noncancer conditions compared with usual care: A randomised controlled single-blind mixed method trial. Int J Nurs Stud 2021; 120: 103978. DOI: 10.1016/j.ijnurstu.2021.103978.

72. Farquhar MC, Prevost AT, McCrone P, et al. Is a specialist breathlessness service more effective and cost-effective for patients with advanced cancer and their carers than standard care? Findings of a mixed-method randomised controlled trial. BMC Med 2014.

73. Farquhar MC, Prevost AT, McCrone P, et al. The clinical and cost effectiveness of a Breathlessness Intervention Service for patients with advanced non-malignant disease and their informal carers: mixed findings of a mixed method randomised controlled trial. Trials 2016.

74. Froggatt K, Best A, Bunn F, et al. A group intervention to improve quality of life for people with advanced dementia living in care homes: the Namaste feasibility cluster RCT. Health Technology Assessment (Winchester, England) 2020; 24: 1–140. DOI: 10.3310/hta24060.

75. Furlan JC, Chan KKW, Sandoval G., et al. An ontario-based cost-utility analysis on the palliative care of patients with metastatic spinal cord cancer: The standard of care versus direct decompressive surgical resection followed by radiotherapy. J Palliat Med 2011.

76. Gottschalk S, König HH, Mallon T, et al. Cost-effectiveness of a specialist palliative care nurse-patient consultation followed by an interprofessional case conference for patients with non-oncological palliative care needs: results of the KOPAL trial. Ann Palliat Med 2023. Article in Press. DOI: 10.21037/apm-23-88.

77. Halling CMB, Wolf RT, Sjogren P, et al. Cost-effectiveness analysis of systematic fast-track transition from oncological treatment to specialised palliative care at home for patients and their caregivers: the DOMUS trial. BMC Palliat Care 2020; 19: 142. DOI: 10.1186/s12904-020-00645-7.

78. Hashimoto Y, Hayashi A, Teng L, et al. Real-World Cost-Effectiveness of Palliative Care for Terminal Cancer Patients in a Japanese General Hospital. J Palliat Med 2021; 24: 1284–1290. DOI: 10.1089/jpm.2020.0649.

79. Huo J, Lairson DR, Du XL, et al. Survival and cost-effectiveness of hospice care for metastatic melanoma patients. Am J Manag Care 2014.

80. Iskedjian M, Iyer S, Librach SL, et al. Methylnaltrexone in the treatment of opioid-induced constipation in cancer patients receiving palliative care: willingness-to-pay and cost-benefit analysis. J Pain Symptom Manage 2011.

81. Jeurnink S, Polinder S, Steyerberg EW, et al. Cost comparison of gastrojejunostomy versus duodenal stent placement for malignant gastric outlet obstruction. J Gastroenterol 2010.

82. Johnson MJ, Kanaan M, Richardson G, et al. A randomised controlled trial of three or one breathing technique training sessions for breathlessness in people with malignant lung disease. BMC Med 2015.

83. Jones L, Fitzgerald G, Leurent B, et al. Rehabilitation in advanced, progressive, recurrent cancer: a randomized controlled trial. J Pain Symptom Manage 2013.

84. Kim H, Rajagopalan MS, Beriwal S, et al. Cost-effectiveness analysis of single fraction of stereotactic body radiation therapy compared with single fraction of external beam radiation therapy for palliation of vertebral bone metastases. Int J Radiat Oncol Biol Phys 2015.

85. Lamfre LS, Hasdeu S, Coller MAG, et al. Economic impact of informal care of cancer patients at the end of life. Ann Palliat Med 2024; 13: 73–85. DOI: 10.21037/apm-23-240.

86. Ljungman D, Hyltander A and Lundholm K. Cost-utility estimations of palliative care in patients with pancreatic adenocarcinoma: a retrospective analysis. World J Surg 2013.

87. Lowery WJ, Lowery AW, Barnett JC, et al. Cost-effectiveness of early palliative care intervention in recurrent platinum-resistant ovarian cancer. Gynecol Oncol 2013.

88. McCaffrey N, Agar M, Harlum J, et al. Is home-based palliative care cost-effective? An economic evaluation of the Palliative Care Extended Packages at Home (PEACH) pilot. BMJ Support Palliat Care 2013.

89. McCaffrey N, Flint T, Kaambwa B, et al. Economic evaluation of the randomised, double-blind, placebo-controlled study of subcutaneous ketamine in the management of chronic cancer pain. Palliat Med 2019.

90. Meads DM, O'Dwyer JL, Hulme CT, et al. Cost-Effectiveness of Pain Management Strategies in Advanced Cancer. Int J Technol Assess Health Care 2019.

91. O'Halloran P, Noble H, Norwood K, et al. Nurse-led advance care planning with older people who have end-stage kidney disease: feasibility of a deferred entry randomised controlled trial incorporating an economic evaluation and mixed methods process evaluation (ACReDiT). BMC Nephrol 2020; 21: 478. DOI: 10.1186/s12882-020-02129-5.

92. Pattenden JF, Mason AR and Lewin RJ. Collaborative palliative care for advanced heart failure: outcomes and costs from the 'Better Together' pilot study. BMJ Support Palliat Care 2013.

93. Pham B and Krahn M. End-of-Life Care Interventions: An Economic Analysis. Ontario Health Technology Assessment Series 2014.

94. Rosato R, Pagano E, Giordano A, et al. Living with severe multiple sclerosis: Cost-effectiveness of a palliative care intervention and cost of illness study. Mult Scler Relat Disord 2021; 49. DOI: 10.1016/j.msard.2021.102756.

95. Sahakyan Y, Li Q, Alibhai SMH, et al. Cost-Utility Analysis of Geriatric Assessment and Management in Older Adults With Cancer: Economic Evaluation Within 5C Trial. J Clin Oncol 2024; 42: 59–69. Article. DOI: 10.1200/JCO.23.00930.

96. Sahlen KG, Boman K. and Brannstrom M. A cost-effectiveness study of person-centered integrated heart failure and palliative home care: Based on a randomized controlled trial. Palliat Med 2016.

97. Sangmala P, Lamlertthon W, Siri P, et al. Economic evaluation of sorafenib treatment of patients with advanced hepatocellular carcinoma at Chulabhorn Hospital. J Med Assoc Thai 2018; 101: S171–S183.

98. Saygili M and Celik Y. An evaluation of the cost-effectiveness of the different palliative care models available to cancer patients in Turkey. Eur J Cancer Care 2019; 28: e13110. DOI: 10.1111/ecc.13110.

99. Sellars M, Clayton JM, Detering KM, et al. Costs and outcomes of advance care planning and end-of-life care for older adults with end-stage kidney disease: A person-centred decision analysis. PLoS One 2019.

100. Shafiq M, Frick KD, Lee H, et al. Management of Malignant Pleural Effusion: A Cost-Utility Analysis. J Bronchology Interv Pulmonol 2015.

101. Suttichaimongkol T, Borntrakulpipat S, Sangchan A, et al. Economic evaluation of palliative biliary drainage in unresectable hilar cholangiocarcinoma. Int J Urol 2018.

102. Thein HH, Qiao Y, Zaheen A, et al. Cost-effectiveness analysis of treatment with non-curative or palliative intent for hepatocellular carcinoma in the real-world setting. PLoS One 2017.

103. Verberkt CA, van den Beuken-van Everdingen MHJ, Dirksen CD, et al. Cost-effectiveness of sustained-release morphine for refractory breathlessness in COPD: A randomized clinical trial. Respir Med 2021; 179: 106330. DOI: 10.1016/j.rmed.2021.106330.

104. Vieira LB, de Barros CM, Price PU, et al. Cost-effectiveness analysis of epidural morphine/ropivacaine treatment in patients with cancer pain. Pain Pract 2024; 24: 25–41. DOI: 10.1111/papr.13278.

105. Wichmann AB, Adang EMM, Vissers KCP, et al. Decreased costs and retained QoL due to the ‘PACE Steps to Success’ intervention in LTCFs: cost-effectiveness analysis of a randomized controlled trial. BMC Med 2020; 18: 258. DOI: 10.1186/s12916-020-01720-9.

106. Wong FKY, So C, Ng AYM, et al. Cost-effectiveness of a transitional home-based palliative care program for patients with end-stage heart failure. Palliat Med 2018.

107. Wu H, Lin P, Yang S, et al. Cost-utility analysis of palliative care in patients with advanced cancer: a retrospective study. BMC Palliat Care 2021; 20: 126. DOI: 10.1186/s12904-021-00816-0.

108. Yi D, Reilly CC, Wei G, et al. Optimising breathlessness triggered services for older people with advanced diseases: A multicentre economic study (OPTBreathe). Thorax 2023; 78: 489–495. Article. DOI: 10.1136/thoraxjnl-2021-218251.
